# Dynamic expectation strength and precision shape human pain perception through shared and dissociable α-oscillatory mechanisms

**DOI:** 10.1371/journal.pbio.3003675

**Published:** 2026-03-02

**Authors:** Jia Li, Shihao Chen, Libo Zhang, Lingling Weng, Xinxin Lin, Yiheng Tu, Weiwei Peng

**Affiliations:** 1 School of Psychology, Shenzhen University, Shenzhen, China; 2 Department of Psychological and Brain Sciences, Dartmouth College, Hanover, New Hampshire, United States of America; 3 State Key Lab of Cognitive Science and Mental Health, Institute of Psychology, Chinese Academy of Sciences, Beijing, China; 4 Department of Psychology, University of Chinese Academy of Sciences, Beijing, China; University of Amsterdam: Universiteit van Amsterdam, NETHERLANDS, KINGDOM OF THE

## Abstract

Human pain perception is not solely driven by sensory input but is dynamically modulated by what we expect to feel and how confident we are in those expectations. Yet, the temporal mechanisms through which evolving expectations shape pain remain poorly understood. Here, we combined a probabilistic cueing paradigm with computational modeling and EEG to dissociate two core components of expectation: strength (a recency-weighted estimate of predicted pain) and precision (the inverse variability of recent predictions). Trial-wise strength estimates closely tracked subjective expectations and outperformed static cue labels, validating the model’s psychological relevance. Expectation strength and precision exerted dissociable effects on pain processing: strength enhanced, whereas precision suppressed, pain-evoked responses. Critically, anticipatory α-band activity mediated these effects via distinct topographical patterns—expectation strength reduced fronto-central α power (reflecting heightened vigilance), while precision increased contralateral sensorimotor α-synchronization (supporting sensory gating). Source-level mediation analyses identified a right-lateralized dorsolateral prefrontal–sensorimotor cortices (DLPFC-SM1) integrating both components, with strength-specific engagement of the medial prefrontal cortex (mPFC). These effects were supported by Bayesian inference and pooled mega-analyses, underscoring their robustness. Together, these findings highlight cortical α-oscillations as dual-control mechanisms for predictive integration, with DLPFC–SM1 as a shared expectation hub and mPFC as a strength-specific node. By moving beyond static cue-based models, this framework captures the adaptive dynamics of expectation and provides a neurocomputational foundation for targeted interventions in chronic pain.

## Abstract 2

Expectations strongly shape pain perception, but how their temporal dynamics influence neural processing is unclear. This study shows that expectation strength and precision have opposite effects on pain-evoked responses, mediated by distinct α‑band mechanisms and prefrontal cortex circuits, revealing a dynamic neural framework for predictive pain modulation.

## Introduction

Human pain perception is not merely a passive reflection of nociceptive input, but a dynamic inferential process shaped by prior experiences and contextual expectations. A growing body of evidence demonstrates that expectations systematically modulate both the subjective experience of pain and its neural correlates [1–5]. This expectation-driven modulation aligns with Bayesian models of perception, where sensory experience emerges from the continuous integration of bottom-up nociceptive input and top-down predictions [6,7]. While prior research has established the importance of anticipatory modulation in pain, critical questions remain regarding the temporal dynamics of expectation formation—namely, how recent sensory outcomes are integrated to shape future predictions and how such dynamically formed expectations influence pain processing.

In natural environments, expectations are not fixed properties of cue identity; instead, they evolve continuously as individuals integrate recent sensory events with prior beliefs. Yet most cue-based pain studies operationalize expectation as a static, categorical variable tied directly to the cue [1,5,8,9]. Such simplifications overlook the inherently dynamic nature of predictive belief updating. Even when cue contingencies are explicitly instructed, probabilistic outcomes still generate meaningful trial-level variability, enabling short-timescale adjustments that reflect ongoing integration of sensory evidence with instructed priors. Predictive coding frameworks emphasize precisely these dynamic updates, positing that expectations emerge from the interplay between top-down priors and bottom-up discrepancies. Although electrophysiological studies have shown that expectations modulate anticipatory α- and β-band activity [1,4,5], their effects on stimulus-evoked pain responses remain inconsistent [1,5,10–12]. These discrepancies may partly reflect conceptual limitations: treating expectation as a dichotomous construct (e.g., “high vs. low”) fails to capture continuous fluctuations in prediction strength based on ongoing sensory feedback [3,13,14], a central principle of predictive coding [7,15–17].

Another limitation of current research is its predominant focus on expectation strength, often overlooking the equally crucial role of expectation precision—the confidence or certainty attached to a prediction. Theoretical frameworks suggest that precision determines the relative influence of top-down expectations versus sensory input [14,18,19]. Empirical findings support this claim, showing that high precision can suppress pain-evoked potentials [14] and reduce neural habituation to predictable stimuli [20,21]. Yet the behavioral consequences of uncertainty vary across studies, with studies reporting both pain amplification [22] and attenuation [23], potentially depending on the expected pain level [24]. These heterogeneous results likely stem from methodological constraints: most studies rely on static predictability metrics or post-hoc confidence ratings that cannot capture trial-level fluctuations in precision. Recent work in pain learning highlights the importance of dynamically modeling belief strength and uncertainty [13,14,25–27], underscoring the need for approaches that jointly quantify both components.

To address these gaps, we implemented a dynamic computational model that estimates trial-by-trial expectations through recency-weighted integration of prior pain experiences. Whereas classical associative-learning models rely on explicit prediction-error computations [4,26–28], the present leaky-integration framework provides a computationally simple and parsimonious characterization of expectation adjustments. Rather than restricting expectation formation to discrete learning events, it captures a continuous predictive process consistent with predictive-coding accounts. Such moment-to-moment adjustments naturally emerge even under instructed probabilistic contingencies, where participants understand the overall cue structure yet continue to recalibrate expectations based on recent experiences. From this model, we derived two key parameters: expectation strength, reflecting the predicted intensity of upcoming pain, and expectation precision, defined as the inverse variability of recent predictions and indexing the certainty of those expectations. This formulation aligns with real-world belief updating, in which stable outcome patterns strengthen predictive confidence whereas outcome variability increases uncertainty and enhances reliance on sensory evidence.

**Fig 1:**
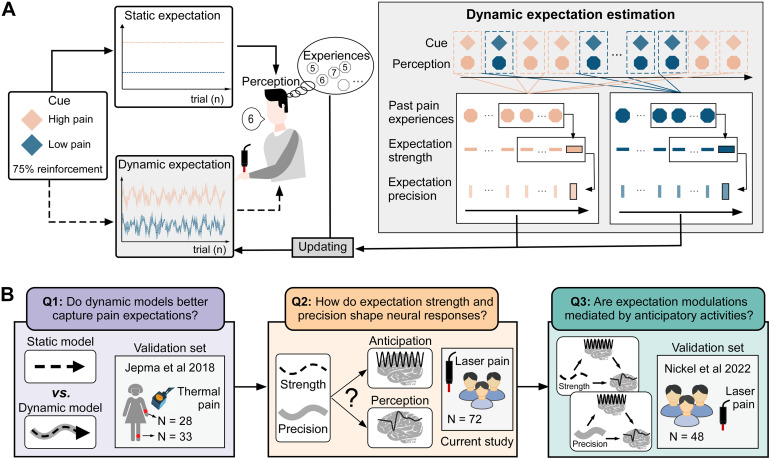
Overview of study design and analytical approach. **(A) Static vs. dynamic models of expectation.** In the static model, expectations are fixed and fully determined by predictive cues, remaining unchanged across trials regardless of experience. In contrast, the dynamic model updates expectations on a trial-by-trial basis via recency-weighted integration of prior pain outcomes. This approach generates continuously evolving estimates of expectation strength (mean predicted pain) and expectation precision (inverse of variance), offering a more ecologically valid account of adaptive expectation formation. **(B) Analytical pipeline addressing three core research questions.** (1) We first compared static cue-based models with a dynamic model to assess their predictive value for subjective pain ratings; (2) Trial-by-trial estimates of expectation strength and precision were used to predict anticipatory EEG activity and pain-evoked responses using Bayesian linear mixed-effects models; (3) Mediation analyses evaluated whether anticipatory neural responses mediated expectation effects on pain, and cross-study validation assessed generalizability using independent datasets.

By treating expectations as continuously evolving rather than static cue-linked quantities, our dynamic model tracks how expectations evolve over time based on recent sensory history (Fig 1A). Combined with EEG recordings, this framework allowed us to probe how dynamic expectations shape both subjective pain and its neural correlates. Based on prior empirical and computational work [1,5,14,23,26,27], we formulated three predefined hypotheses corresponding to our planned analyses: (1) that dynamic, trial-by-trial computational models would provide a more accurate account of pain-related expectations than static cue-based approaches; (2) that expectation strength and precision would exert dissociable influences on anticipatory and stimulus-evoked neural responses; (3) that these modulatory effects would be implemented through specific large-scale oscillatory dynamics. These three planned questions formed the core of our confirmatory analyses and structured the analytical pipeline of the study (Fig 1B). Additional analyses—such as extended model comparisons and cross-dataset robustness checks—provided further mechanistic insight and validated generalizability.

Drawing on the primary dataset collected for the present study, and complemented by a harmonized re-analysis of an external dataset from Nickel and colleagues 2022 [5], we identified robust and generalizable patterns linking dynamic expectations to anticipatory α-band oscillations. Specifically, fronto-central α desynchronization scaled with expectation strength, contralateral sensorimotor α synchronization tracked expectation precision. Source-level analyses further revealed that a right-lateralized dorsolateral prefrontal–sensorimotor network jointly encoded both expectation parameters, while medial prefrontal α oscillations selectively reflected expectation strength. These findings advance a neurocomputational framework of pain processing, in which the content and precision of dynamic expectations are encoded via dissociable α-band mechanisms—bridging predictive coding theories with empirical neural data. By identifying α rhythms as a flexible gain-control system for top-down modulation, this work highlights potential targets for disrupting maladaptive expectations and improving pain management in clinical settings.

## Results

### Validation of expectation manipulation

**Fig 2:**
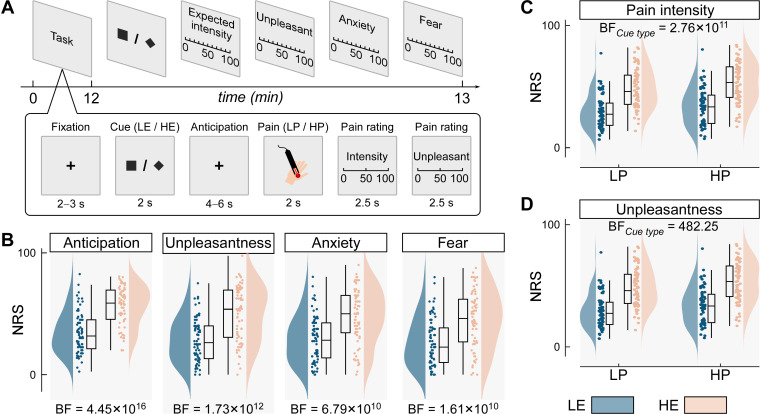
Experimental design and validation of expectation manipulation. **(A) Task structure and trial timeline.** The top panel illustrates the overall experimental protocol, and the bottom panel depicts the sequence of events within a single trial. Each trial began with a fixation cross, followed by a predictive visual cue (diamond or square). After a jittered anticipation interval (4–6 s), a brief laser stimulus—either low (LP) or high (HP) in intensity—was delivered to the dorsum of the left hand. Participants then verbally rated perceived pain intensity and unpleasantness. At the end of each run, participants provided offline ratings of expected pain intensity, unpleasantness, anxiety, and fear associated with each cue type. Cue–pain contingencies were counterbalanced across participants: for half the sample, diamond cues predicted LP stimuli with 75% probability (low-expectation, LE cues), and square cues predicted HP stimuli (high-expectation, HE cues); these assignments were reversed in the other half. **(B) Offline cue ratings confirming successful expectation manipulation.** HE cues were associated with significantly higher ratings of expected pain intensity and unpleasantness, as well as greater anticipatory anxiety and fear, compared to LE cues. **(C–D) Online pain ratings.** Participants reported higher pain intensity **(C)** and unpleasantness **(D)** when trials were preceded by HE cues, regardless of actual stimulus intensity. Cloud plots show the distribution of individual means, overlaid with boxplots indicating the median and interquartile range (IQR, Q1–Q3); whiskers extend to 1.5 × the IQR. BF: Bayes factor. Values > 10 indicate strong evidence for cue-related effects. Data supporting this figure are available at https://doi.org/10.5281/zenodo.18503056.

We recorded EEG data from 72 participants (35 females) using a modified cued-pain paradigm designed to probe dynamic expectation profiles. The paradigm was adapted from prior work [5,8], presenting two visual cues: a high-expectation (HE) cue predominantly paired with high-intensity laser pain (HP) and a low-expectation (LE) cue paired with low-intensity pain (LP), each with a 75% reinforcement probability. Following each laser stimulus, participants rated perceived pain intensity and unpleasantness on a 0–100 numerical rating scale (NRS; 0 = “no pain” or “no unpleasantness,” 100 = “unbearable pain sensation” or “extremely unpleasant”). At the end of each run, they also rated each cue’s associated expected pain intensity, unpleasantness, anxiety, and fear using the same NRS. The experiment comprised 160 trials across four runs (Fig 2A).

To validate the cue–pain contingency, we conducted Bayesian paired-sample *t*-tests comparing offline ratings between HE and LE cues. Bayes factors (BFs) were calculated to quantify evidence in favor of the alternative (H₁) versus null (H₀) hypotheses. BFs > 3 (or < 0.33) and > 10 (or < 0.1) were interpreted as moderate and strong evidence for or against H₁, respectively [29]. We found decisive evidence for higher ratings for HE versus LE cues across all measures: expected pain intensity (BF = 4.45 × 10^16^), unpleasantness (BF = 1.73 × 10^12^), anxiety (BF = 6.79 × 10^10^), and fear (BF = 1.61 × 10^10^). These values provide extreme evidence for the effectiveness of the expectation manipulation (Fig 2B).

To assess whether these cue-based expectations influenced actual pain experience, we conducted Bayesian repeated-measures analyses of variance (rmANOVAs) with stimulus intensity (HP vs. LP) and cue type (HE vs. LE) as within-participant factors. We observed extreme evidence for main effects of both stimulus intensity (BFs ≥ 4.62 × 10^14^) and cue type (BFs ≥ 482.25) on pain intensity and unpleasantness ratings, with no conclusive evidence for an interaction (BFs ≤ 2.42, indicating inconclusive evidence between the hypotheses). This pattern replicates the findings of Nickel and colleagues 2022 [5] and supports a predictive coding account in which both bottom-up nociceptive input and top-down expectations jointly shape pain perception (Fig 2C*–*2D).

### Computational modeling of dynamic expectation

**Fig 3:**
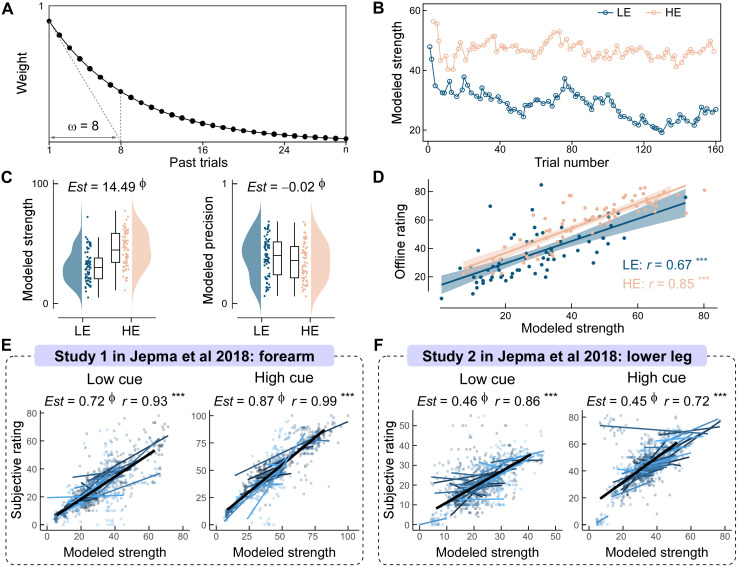
Computational modeling and validation of dynamic expectations. **(A) Temporal weighting in the dynamic expectation model.** Expectation strength was modeled as a weighted sum of past pain experiences, with recent trials (≤ 8) weighted highest due to memory decay. **(B) Example trajectory of modeled expectation strength.** A representative participant exhibited sustained divergence in expectation strength between high-expectation (HE) and low-expectation (LE) cues across trials. **(C) Group-level estimates of expectation strength and precision by cue type.** HE cues elicited higher expectation strength and lower precision compared to LE cues (Bayesian linear mixed-effects model: *Expectation strength/Precision ~ Cue + (1 | Participant)*). Boxplots denote medians, interquartile ranges (IQR, Q1–Q3), and whiskers extend to 1.5 × IQR. Cloud plots indicate the distribution of individual means. **(D) Convergent validity with self-reported expectations.** Modeled expectation strength correlated significantly with subjective ratings across both cue conditions. **(E–F) Model validation using independent data from Jepma and colleagues 2018** [3]. Gray dots represent individual data; thick colored lines indicate participant-level fits; black lines represent group trends. Modeled and self-reported expectation strength showed robust positive correlations across datasets and cue types. ***p < 0.001; Ф: posterior probability > 97.5% and 95% highest probability density excluding 0. Data supporting this figure are available at https://doi.org/10.5281/zenodo.18503056.

To quantify how pain expectations evolve over time, we implemented a computational model based on leaky integration principles, in which recent experiences are given greater weight than more temporally distant events. Specifically, the model computed expectation strength as the exponentially weighted average of prior pain intensity, with weights defined by the function $e^{-k/\omega \;}$ [14,23], where *k* represents trial recency and *ω* = 8 denotes the memory decay parameter (Fig 3A). Expectation precision was derived as the inverse of the weighted variance of strength estimates. Both parameters were normalized to the [0,1] interval for statistical analysis. A representative trajectory (Fig 3B) demonstrated sustained divergence in expectation strength following HE versus LE cues, consistent with the anticipated manipulation, while precision dynamically adapted based on trial-wise variance.

To validate the sensitivity of the model to experimental manipulations, we applied Bayesian linear mixed-effects models (LMMs) with cue type (LE/HE) as a fixed factor. Robust cue-related differences were found in both strength and precision of expectation (Fig 3C). Expectation strength was robustly higher following HE compared to LE cues (Estimate [*Est*] = 14.49, 95% highest probability density [HPD] = [14.15, 14.81], posterior probability [*P*p] = 1.00), while precision was compellingly lower following HE cues (*Est* = −0.02, 95% HPD = [−0.03, −0.02], *P*p = 1.00), indicating increased uncertainty under high-pain anticipation.

To assess the construct validity of model-derived expectations, we examined their correlation with participants’ self-reported expectation ratings collected offline after each experimental run. For each cue type, we computed between-participant Pearson correlations between averaged model-derived strength values and subjective ratings. Strong positive correlations were observed for both LE cues (*r* = 0.67, *p* < 0.001) and HE cues (*r* = 0.85, *p* < 0.001; Fig 3D), supporting the high fidelity of the computational estimates.

To evaluate the validity of the model, we applied the same computational approach to two independent datasets from Jepma and colleagues 2018 [3], in which participants rated their trial-by-trial expected pain intensity prior to stimulation. Bayesian LMMs showed strong associations between model-derived and subjective expectation strength in both studies: for Study 1 (forearm stimulation), the estimated effect was 0.72 (95% HPD = [0.59, 0.84]) under LE cues and 0.87 (95% HPD = [0.69, 1.02]) under HE cues; for Study 2 (leg stimulation), estimates were 0.46 (95% HPD = [0.30, 0.61]) and 0.45 (95% HPD = [0.31, 0.60]), respectively, all with *P*p = 1.00 (Fig 3E–3F). At the participant level, between-participant Pearson correlations further confirmed robust associations across cue types and studies (all *r* ≥ 0.72, all *p* < 0.001), reinforcing the external validity of the model.

Finally, to determine whether dynamic expectations outperform static cue labels in explaining subjective expectation strength, we conducted a formal model comparison. We constructed Bayesian LMMs predicting subjective expectation using either model-derived strength or cue type (LE/HE). Model comparisons via Bayes factor analysis (R package BayesFactor, version 0.9.12; [30]) revealed decisive evidence in favor of the dynamic model (Current study: BF = 1.17 × 10¹⁷; Jepma and colleagues 2018: BF = 7.03 × 10¹⁶), indicating that trial-by-trial updates in expectation strength provided a superior account of participants’ self-reported beliefs than did the static cue identity alone.

Together, these findings confirm that the leaky integration model provides a valid and generalizable quantification of dynamic pain expectations. By capturing the trial-wise evolution of both expectation strength and precision, this approach enables fine-grained analyses of how expectations shape perception and brain activity beyond conventional static models. Moreover, formal model comparisons using the VBA toolbox [31], against well-established prediction-error based learning frameworks (including Rescorla–Wagner and Bayesian Kalman-filter models) in both our dataset and the independent datasets from Jepma and colleagues 2018 [3] showed that the leaky integration model consistently offered superior explanatory power for participants’ actual expectations (see Text C, Table B, and Figs B, C in S1 File).

### Effect of dynamic expectation on pain-related responses

Having validated our computational model of dynamic expectation, we next examined how expectation strength and precision modulated subjective pain perception and its underlying neural correlates. We focused on three core pain-related measures: (1) behavioral pain ratings (intensity and unpleasantness), (2) time-domain laser-evoked potentials (LEPs; N2 and P2 components), and (3) time–frequency neural oscillatory responses (LEP magnitude, α-event-related desynchronization [α-ERD], and γ-event-related synchronization [γ-ERS]). Across all analyses, we employed Bayesian LMMs, with fixed effects for stimulus intensity, expectation strength, precision, and their interactions, and random intercepts for participant.

**Fig 4:**
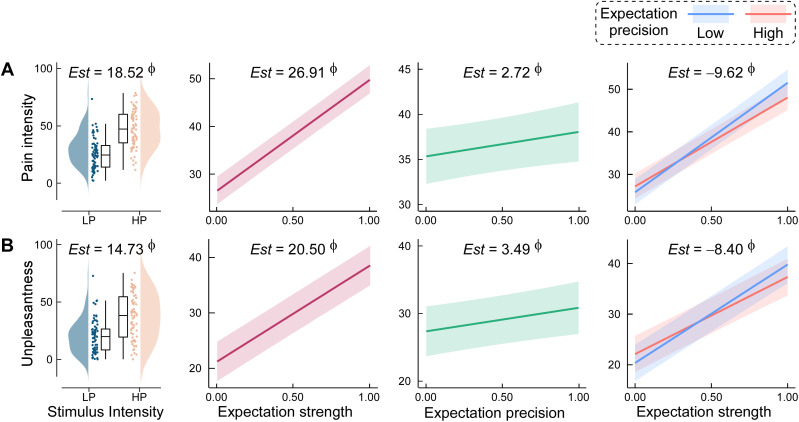
Dynamic expectation effects on pain ratings. Effects of dynamic expectation on perceived pain ratings. Bayesian linear mixed-effects models incorporating trial-by-trial expectation strength and precision—alongside stimulus intensity—revealed that both parameters modulated **(A)** pain intensity and **(B)** unpleasantness ratings. Estimated slopes (*Est*s) and Bayesian evidence are shown; Φ indicates strong evidence (posterior probability > 97.5%, with 95% highest probability density excluding 0). Boxplots indicate medians, interquartile ranges (IQR, Q1–Q3), and whiskers extend to 1.5 × IQR. Cloud plots show the distribution of individual means. Shaded bands represent 95% prediction intervals. Data supporting this figure are available at https://doi.org/10.5281/zenodo.18503056.

Behavioral data revealed robust effects of both stimulus and expectation profiles on pain perception (Fig 4). As expected, higher stimulus intensity robustly increased ratings of pain intensity (*Est* = 18.52, 95% HPD = [17.91, 19.14], *P*p = 1.00) and unpleasantness (*Est* = 14.73, 95% HPD = [14.11, 15.34], *P*p = 1.00). Expectation strength also strongly enhanced both intensity (*Est* = 26.91, 95% HPD = [24.71, 29.11], *P*p = 1.00) and unpleasantness (*Est* = 20.50, 95% HPD = [18.36, 22.64], *P*p = 1.00) ratings. Precision showed modest yet credible positive effects (intensity: *Est* = 2.72, 95% HPD = [0.28, 5.10], *P*p = 0.99; unpleasantness: *Est* = 3.49, 95% HPD = [1.17, 5.82], *P*p = 1.00). Notably, we observed compelling evidence for the interaction between strength and precision (intensity: *Est* = −9.62, 95% HPD = [−14.17, −5.03], *P*p = 1.00; unpleasantness: *Est* = −8.40, 95% HPD = [−12.77, −3.92], *P*p = 1.00), indicating that the modulatory impact of expectation strength on pain was amplified under low-precision conditions. This pattern suggests that metacognitive uncertainty enhances the perceptual influence of top-down predictions. To ensure that model-derived expectations were not circular reflections of stimulus intensity or gradual sensory habituation, all primary models explicitly controlled for stimulus intensity, and supplementary validation analyses showed that expectation strength and precision remained robust predictors even after accounting for cue validity and trial repetition (see Table C and Text D in S1 File).

**Fig 5:**
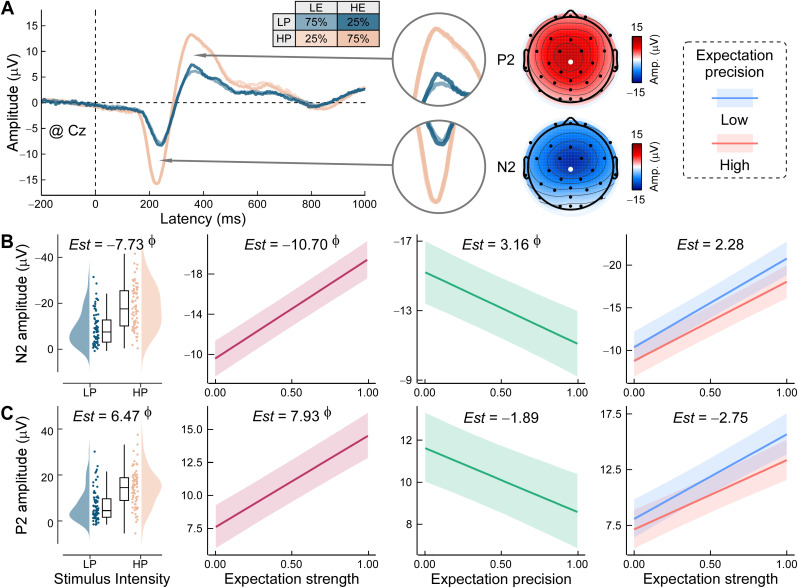
Dynamic expectation effects on laser-evoked potentials (LEPs). **(A) Grand-averaged LEPs across stimulus and expectation conditions.** Waveforms are plotted for all combinations of stimulus intensity (high pain, HP; low pain, LP) and cue-based expectation (high, HE; low, LE). Scalp topographies show the spatial distribution of the N2 and P2 components, with Cz electrode marked by a white dot. **(B–C) Effects of dynamic expectation on N2 and P2 amplitudes.** Bayesian linear mixed-effects models tested the influence of stimulus intensity, expectation strength, precision, and their interactions on the amplitude of **(B)** N2 and **(C)** P2 components. Estimated slopes (*Est*s) and corresponding Bayesian evidence are reported. Φ denotes strong effects (posterior probability > 97.5%, with 95% highest probability density excluding 0). Boxplots show medians, interquartile ranges (IQR, Q1–Q3), and whiskers extend to 1.5 × IQR. Cloud plots indicate the distribution of individual means. Shaded bands represent 95% prediction intervals. BF: Bayes factor. Data supporting this figure are available at https://doi.org/10.5281/zenodo.18503056.

To characterize the temporal dynamics of expectation effects, we analyzed the amplitude of LEP components at the Cz electrode (Fig 5A). As anticipated, both N2 and P2 were strongly modulated by stimulus intensity (N2: *Est* = −7.73, 95% HPD = [−8.27, −7.20], *P*p = 1.00; P2: *Est* = 6.47, 95% HPD = [5.95, 6.99], *P*p = 1.00). Expectation strength also robustly influenced both components (N2: *Est* = −10.70, 95% HPD = [−12.59, −8.81], *P*p = 1.00; P2: *Est* = 7.93, 95% HPD = [6.11, 9.75], *P*p = 1.00), consistent with enhanced salience under high-expectation states. Importantly, precision showed a credible positive effect on N2 amplitude (*Est* = 3.16, 95% HPD = [1.10, 5.22], *P*p = 1.00; Fig 5B), suggesting reduced early nociceptive responses when expectations are more precise. P2 modulation by precision was weaker and not robust (*Est* = −1.89, 95% HPD = [−3.89, 0.10], *P*p = 0.97; Fig 5C). These results highlight temporally dissociable effects of expectation profiles on early and late stages of pain-evoked cortical processing.

**Fig 6:**
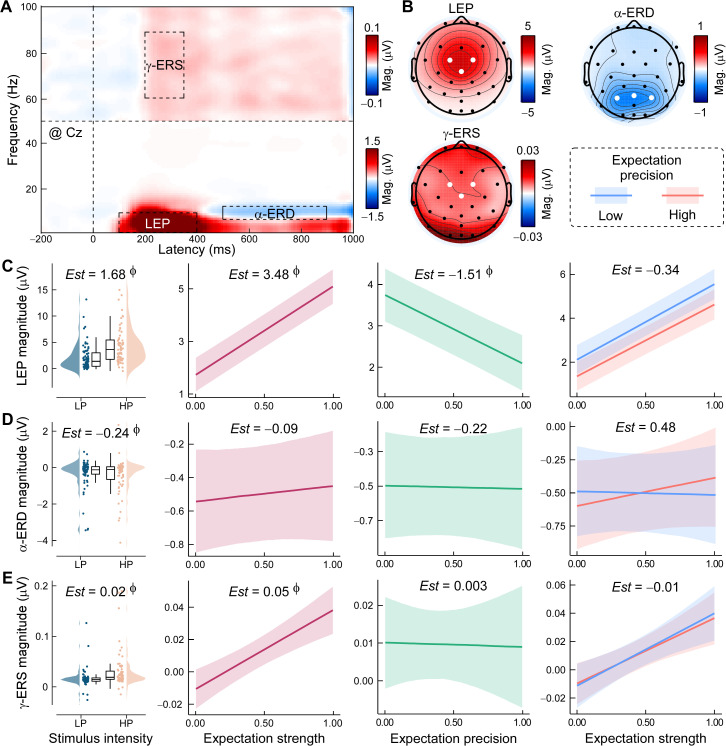
Dynamic expectation effects on laser induced neural oscillations. **(A) Time–frequency representations (TFRs) of pain-related neural oscillations.** Grand-averaged TFRs at Cz depict baseline-corrected power changes (relative to a −400 to −100 ms pre-stimulus interval). Dotted boxes highlight regions of interest: laser-evoked potentials (LEPs), alpha-band event-related desynchronization (α-ERD), and gamma-band event-related synchronization (γ-ERS). **(B) Topographical distributions of oscillatory responses.** Grand-averaged scalp topographies show the spatial distribution of each neural response, with white dots indicating electrodes of maximal magnitude. **(C–E) Bayesian linear mixed-effects models assessing dynamic expectation effects.** Stimulus intensity, expectation strength, precision, and their interactions were modeled as predictors of **(C)** LEP amplitude, **(D)** α-ERD magnitude, and **(E)** γ-ERS magnitude. Estimated slopes (*Est*s) and Bayesian evidence are reported. Φ denotes strong effects (posterior probability > 97.5%, with 95% highest probability density excluding 0). Boxplots show medians and interquartile ranges (IQR, Q1–Q3), with whiskers extending to 1.5 × IQR. Cloud plots represent the distribution of individual means. Shaded bands reflect 95% prediction intervals. Data supporting this figure are available at https://doi.org/10.5281/zenodo.18503056.

Time–frequency analyses further revealed distinct oscillatory responses associated with pain and expectation (Fig 6A–6B). LEP magnitude was robustly increased by both stimulus intensity (*Est* = 1.68, 95% HPD = [1.50, 1.85], *P*p = 1.00) and expectation strength (*Est* = 3.48, 95% HPD = [2.86, 4.10], *P*p = 1.00), but reduced by precision (*Est* = −1.51, 95% HPD = [−2.20, −0.82], *P*p = 1.00; Fig 6C). α-ERD was primarily driven by stimulus intensity (*Est* = −0.24, 95% HPD = [−0.38, −0.09], *P*p = 1.00; Fig 6D) with no reliable modulation by expectation profiles, suggesting it mainly reflects sensory-driven cortical arousal. Conversely, γ-ERS was compellingly enhanced by expectation strength (*Est* = 0.05, 95% HPD = [0.02, 0.09], *P*p = 1.00; Fig 6E), consistent with greater attentional engagement or salience encoding during high-expectancy trials.

Together, these findings demonstrate that dynamic expectations shape pain-related responses across multiple temporal and spectral domains. Expectation strength consistently amplifies both subjective experience and neural reactivity, whereas precision functions as a modulatory filter—dampening early nociceptive processing and adjusting the gain of top-down predictive signals under conditions of uncertainty.

When expectations were modeled categorically (low vs. high), we observed strong main effects of both stimulus intensity (BFs ≥ 4.62 × 10¹⁴) and expectation (BFs ≥ 482.25) on pain ratings, but no interaction (BFs ≤ 2.42; see Fig 2 and Table A in S1 File). Neural responses, however, were driven mainly by stimulus intensity (BFs ≥ 24.28), with no reliable effects of expectation (BFs ≤ 0.90; see Text A, Table A, and Fig A in S1 File). This replicates previous findings (Nickel and colleagues 2022 [5]) and illustrates a key limitation of static cue-based designs: they conflate external cues with participants’ internal expectations, masking finer neural effects. In contrast, our continuous, computational approach offers a more sensitive and ecologically valid way to capture how dynamic expectations shape pain. These findings highlight the value of trial-wise modeling in uncovering subtle brain–behavior links often missed by conventional analyses.

### Effect of dynamic expectation on anticipatory EEG oscillations

**Fig 7:**
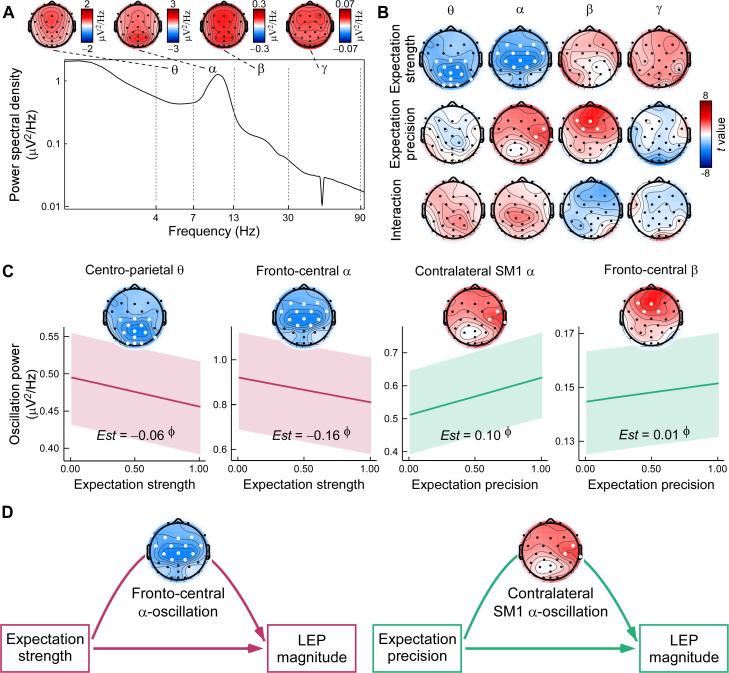
Dynamic expectation effects on anticipatory EEG oscillations. **(A) Spectral dynamics during pain anticipation.** Top: Scalp topographies show band-averaged power across the anticipation window, with dotted lines indicating frequency boundaries. Bottom: Grand-averaged power spectral density at Cz, visualized on a log–log scale, reveals activity in the θ (4–7 Hz), α (7–13 Hz), β (13–30 Hz), and γ (30–90 Hz) bands during pain anticipation. **(B) Whole-brain linear mixed-effects analyses of expectation strength and precision.** T-statistic maps display fixed effects from the model (*Power ~ Expectation Strength × Precision + (1 | Participant)*), with FDR-corrected electrodes highlighted (bold white). Expectation strength modulated centro-parietal θ and fronto-central α power, while precision influenced contralateral sensorimotor α and fronto-central β power. **(C) Bayesian post hoc analysis of key oscillatory responses.** Estimated slopes (*Est*s), and 95% prediction intervals (shaded)are shown. Φ denotes strong evidence (posterior probability > 97.5%, with 95% highest probability density excluding 0). **(D) Mediation models linking expectation, anticipatory EEG oscillation, and pain-evoked responses.** Fronto-central α power mediated the effect of expectation strength on LEP magnitude, while contralateral sensorimotor α power mediated precision-related effects. Data supporting this figure are available at https://doi.org/10.5281/zenodo.18503056.

We next examined how dynamic expectation profiles modulated anticipatory neural activity during the pain anticipation period. Spectral characteristics and scalp topographies of the anticipatory EEG signals are shown in Fig 7A. To capture spatially distributed frequency-specific modulations, we conducted whole-brain LMMs across four canonical frequency bands: theta (θ, 4–7 Hz), alpha (α, 7–13 Hz), beta (β, 13–30 Hz), and gamma (γ, 30–90 Hz). Each model included Expectation strength, precision, and their interaction as fixed effects, with participant-level random intercepts. Results were corrected for multiple comparisons using the false discovery rate (FDR) method.

As shown in Fig 7B, distinct frequency–spatial modulation patterns emerged. Expectation strength was associated with reduced θ power over centro-parietal regions (e.g., C3, Cz, CP1, Pz) and reduced α power over fronto-central electrodes (e.g., Fz, FC1, Cz). In contrast, expectation precision was associated with increased α power over contralateral sensorimotor sites (e.g., FC6, C4, T8) and increased β power in fronto-central regions (e.g., Fz, FC2). *Post-hoc* Bayesian LMMs further confirmed these patterns (Fig 7C). Specifically, expectation strength negatively predicted both centro-parietal θ power (*Est* = −0.06, 95% HPD = [−0.09, −0.03], *P*p = 1.00) and fronto-central α power (*Est* = −0.16, 95% HPD = [−0.24, −0.08], *P*p = 1.00), suggesting increased cognitive vigilance or attentional engagement under higher expectations. In contrast, expectation precision positively predicted contralateral sensorimotor α power (*Est* = 0.10, 95% HPD = [0.04, 0.16], *P*p = 1.00) and fronto-central β power (*Est* = 0.01, 95% HPD = [0.01, 0.02], *P*p = 1.00), indicating enhanced preparatory or inhibitory control under greater certainty. Further exploratory time-frequency analyses observed similar patterns across the anticipation interval (see Text E and Fig D in S1 File).

### Mediation models of expectation-driven pain modulation

To investigate the oscillatory mechanisms through which dynamic expectations shape pain perception, we conducted multilevel mediation analyses linking trial-wise expectation parameters, anticipatory brain dynamics, and pain-evoked responses. This analysis addressed whether the effects of expectation strength and precision on pain responses were mediated by specific oscillatory signatures during anticipation, and whether such mechanisms were reproducible across independent datasets.

At the sensor level, anticipatory EEG activity significantly mediated the relationship between expectation and pain-evoked responses (Fig 7D). Specifically, expectation strength exerted a positive indirect effect on LEP magnitude via fronto-central α power suppression (indirect effect = 0.003, *p*_FDR_ < 0.001, FDR-adjusted *p* value), indicating that stronger expectations reduced anticipatory α oscillations, thereby enhancing subsequent nociceptive responses. In contrast, expectation precision exhibited a negative indirect effect (indirect effect = −0.002, *p*_FDR_ = 0.02), such that higher precision increased contralateral sensorimotor α power, which in turn dampened LEP magnitude. These dual dissociations suggest that while expectation strength facilitates pain through cortical disinhibition, precision attenuates it via anticipatory gating.

**Fig 8:**
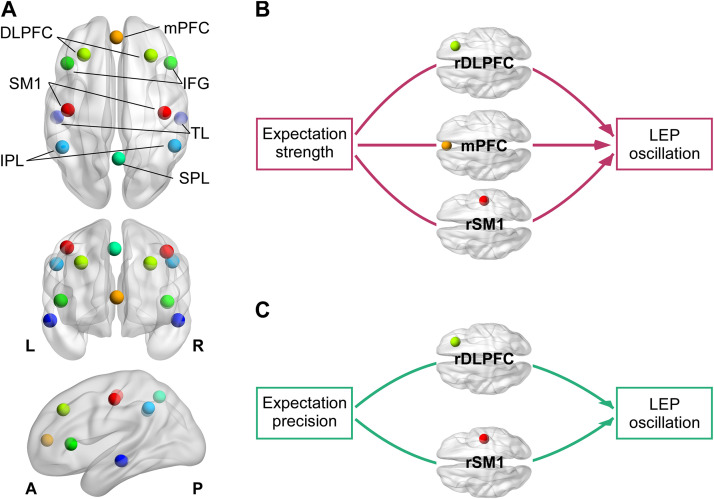
Neural mechanisms of dynamic expectation-driven pain modulation. **(A) Regions of interest (ROIs).** Anatomical locations of 12 predefined ROIs are shown across axial, sagittal, and coronal planes. **(B–C) Source-level mediation models linking expectation, brain oscillations, and laser-evoked potentials (LEPs). (B)** Trial-by-trial expectation strength modulated LEP magnitude via anticipatory α-oscillatory activity in the right dorsolateral prefrontal cortex (DLPFC), contralateral primary sensorimotor cortex (SM1), and medial prefrontal cortex (mPFC). **(C)** Expectation precision exerted indirect effects on LEP magnitude via α power in the right DLPFC and contralateral SM1. Abbreviations: mPFC, medial prefrontal cortex; DLPFC, dorsolateral prefrontal cortex; SM1, primary sensorimotor cortex; IFG, inferior frontal gyrus; TL, temporal lobe; IPL, inferior parietal lobule; SPL, superior parietal lobule; L, left; R, right; A, anterior; P, posterior. Data supporting this figure are available at https://doi.org/10.5281/zenodo.18503056.

To identify the cortical sources of these oscillatory mediators, we performed source-level analysis using FieldTrip across 12 predefined regions of interest (ROIs; see Fig 8A and Table D in S1 File) encompassing medial prefrontal cortex (mPFC), dorsolateral prefrontal cortex (DLPFC), primary sensorimotor cortex (SM1), inferior frontal gyrus (IFG), inferior and superior parietal lobule (IPL, SPL), and temporal lobe (TL). LMMs with FDR correction revealed distinct spatial patterns: expectation strength was associated with α power suppression in bilateral DLPFC, SM1, and midline mPFC, whereas expectation precision selectively enhanced α activity in right-lateralized regions, including rDLPFC, rSM1, and rIFG—contralateral to the site of stimulation. Bayesian models (Table 1) confirmed these effects (strength: *Est*s ≤ −0.058, *P*ps ≥ 0.996; precision: *Est*s ≥ 0.090, *P*ps = 1.00), supporting a functional dissociation between strength- and precision-driven circuits.

**Table 1:** Statistics of the Bayesian LMMs on source-level α-band oscillations within predefined ROIs.

ROIs	Expectation strength			Expectation precision			Expectation strength × precision		
	*Est*	95% HPD	*P*p	*Est*	95% HPD	*P*p	*Est*	95% HPD	*P*p
lSM1	−**0.079**	**[**−**0.132,** −**0.025]**	**0.998**	0.049	[−0.010, 0.106]	0.947	0.078	[−0.032, 0.189]	0.918
rSM1	−**0.102**	**[**−**0.168,** −**0.034]**	**0.999**	**0.141**	**[0.067, 0.215]**	**1.000**	0.009	[−0.133, 0.148]	0.553
mPFC	−**0.110**	**[**−**0.189,** −**0.031]**	**0.996**	**0.116**	**[0029, 0.204]**	**0.995**	−0.018	[−0.182, 0.147]	0.582
lDLPFC	−**0.071**	**[**−**0.105,** −**0.036]**	**1.000**	0.031	[−0.007, 0.069]	0.948	0.063	[−0.008, 0.135]	0.959
rDLPFC	−**0.058**	**[**−**0.099,** −**0.017]**	**0.997**	**0.090**	**[0.044, 0.134]**	**1.000**	−0.026	[−0.112, 0.059]	0.723
rIFG	−0.019	[−0.075, 0.039]	0.739	**0.147**	**[0.085, 0.211]**	**1.000**	−**0.144**	**[**−**0.265,** −**0.023]**	**0.992**
rTL	−0.088	[−0.270, −0.094]	0.825	**0.421**	**[0.224, 0.621]**	**1.000**	−0.275	[−0.653, 0.101]	0.923

Bayesian linear mixed-effects model (LMM) with the formula: *DV ~ Expectation strength × Precision + (1 | Participant)*. Estimated slopes (*Est*s) were considered statistically compelling when posterior probability (*P*p) > 97.5%, and 95% highest probability density (HPD) interval excluded 0. Robust effects meeting these criteria are marked in bold. Abbreviations: DV, Dependent variable. Brain regions: lSM1/rSM1, Left/right primary sensorimotor cortex; mPFC, Medial prefrontal cortex; lDLPFC/rDLPFC, Left/right dorsolateral prefrontal cortex; rIFG: Right inferior frontal gyrus; rTL: Right temporal lobe. Data supporting this table are available at https://doi.org/10.5281/zenodo.18503056.

Further source-level mediation analyses (FDR-corrected; Fig 8B–8C) demonstrated that right DLPFC, contralateral SM1, and mPFC were key hubs in these expectation-induced pain modulations. Specifically, reduced α activity in these regions mediated the facilitatory effect of expectation strength on LEP magnitude (indirect effects ≥ 0.0014, *p*_FDR_s ≤ 0.03), while enhanced α in right DLPFC and SM1 mediated the attenuating effect of precision (indirect effects ≤ −0.002, *p*_FDR_s ≤ 0.03). These results suggest a computational specialization: the dorsolateral prefrontal and somatosensory cortices encode precision-weighted expectations, while the mPFC uniquely tracks the motivational salience associated with expectation strength.

### Robustness and generalizability assessment via mega-analysis

To evaluate the robustness and generalizability of these findings, we conducted pooled mega-analyses [32,33] combining data from the current study and Nickel and colleagues 2022 [5], while accounting for potential heterogeneity across datasets. Results reinforced all core mechanisms across datasets (see Text G and Figs F–I in S1 File): (1) Subjective pain ratings were jointly shaped by stimulus intensity, expectation strength, and precision (all with *P*p = 1.00); (2) Pain-evoked neural responses, including N2–P2 and LEP magnitudes were modulated by both bottom-up sensory input and top-down expectations—strength amplified while precision attenuated neural reactivity; (3) Anticipatory oscillations exhibited frequency- and location-specific dissociations: strength suppressed θ/α activity while precision enhanced α/β activity, with distinct oscillatory mechanisms confirming the functional relevance of these dynamics. Collectively, these findings delineate a robust and replicable neurocognitive mechanism by which dynamic expectations—differentiated by strength and precision—modulate pain perception via distinct anticipatory brain states.

## Discussion

Our findings show that dynamically modeled expectations—computed trial-by-trial from recent pain experiences—outperform static cue-based models in capturing subjective pain expectations. Critically, we dissociated two key components: expectation strength and precision. Strength amplified pain-evoked responses (LEPs and γ-oscillations), while precision attenuated nociceptive encoding. These effects were mediated by distinct anticipatory α mechanisms: strength suppressed fronto-central α power (suggesting heightened vigilance), whereas precision enhanced contralateral sensorimotor α synchronization (indicating sensory gating). Source analyses revealed that both components converged on α-mediated mechanisms: a right-lateralized DLPFC–SM1 circuit integrated both strength and precision, mPFC uniquely orchestrated strength-specific α-modulation. Importantly, these neurocomputational mechanisms were replicated in independent datasets, supporting their generalizability. Together, our findings position cortical α rhythms as predictive integrators in pain processing, highlight the DLPFC–SM1 circuit as a multidimensional expectation hub, and identify the mPFC as a potential therapeutic target for disrupting maladaptive predictions.

### Expectation-induced modulation of pain responses

Our leaky integration model offers a conceptually grounded and empirically robust framework for characterizing dynamic pain expectations [14,23,34]. By simulating trial-by-trial updates of expectation strength and precision through recency-weighted integration of previous outcomes, the model captures key features of naturalistic learning overlooked by traditional static cue-based approaches. Crucially, trial-wise estimates of expectation strength derived from this model showed strong correlations with participants’ subjective reports of expected pain intensity across both high- and low-expectancy conditions. These correlations were robust not only in our primary dataset but also in two independent replication datasets, demonstrating the model’s generalizability. Moreover, Bayesian model comparisons confirmed that dynamic expectation strength explained significantly more variance in subjective ratings than cue identity alone. This converges with prior work showing that cue–outcome associations are continuously updated [4,26,28], reinforcing the view that internal pain-prediction models are not fixed representations but evolve through ongoing sensory evidence [3,35]. Together, these findings establish dynamic modeling as a powerful approach for quantifying belief updating under uncertainty.

Building on this validated framework, we next examined how trial-wise fluctuations in expectation strength and precision influenced subjective pain perception and its underlying neural correlates. Consistent with earlier work [1,5,10–12,36], both subjective ratings (intensity, unpleasantness) and neural responses to laser pain (LEPs, α-ERD, γ-ERS) were dominated by stimulus intensity, underscoring the primacy of bottom-up nociceptive input across temporal and spectral scales. However, our dynamic model revealed a functional dissociation between expectation profiles: strength amplified nociceptive processing, while precision attenuated it. Specifically, greater expectation strength amplified nociceptive processing at multiple levels—enhancing LEP responses (N2 and P2) and midfrontal γ-oscillations—whereas precision selectively dampened LEP responses, reducing N2 and, to a lesser extent, P2 amplitudes.

Expectation strength appears to function as a gain-control signal, amplifying nociceptive signals across processing stages [10–12,36–38]. Expectation strength bidirectionally modulated early sensory processing stages, as evidenced by gain control effects on the N2 component—associated with nociceptive salience detection [38,39]—and the subsequent P2 component reflecting perceptual evaluation [38,40]. Strength likewise elevated γ-band oscillations, which coordinate distributed cortical networks during conscious perception [5,11]. This highlights its role in prioritizing high-strength predictions for conscious integration [41,42], potentially optimizing adaptive responses to anticipated pain stimuli [43]. In contrast, expectation precision demonstrated a distinct suppression profile: higher precision attenuated both N2 amplitudes (salience detection) and P2 amplitudes (perceptual evaluation), with marginal effects on the latter. This dual-stage attenuation challenges purely sensory-level accounts of precision’s effects, instead suggesting it implements predictive filtering across sequential processing phases. Mechanistically, this may reflect precision-weighted gating of sensory evidence accumulation [14,20,44], where precise priors reduce prediction error signaling not just at initial encoding (N2) but also later perceptual consolidation (P2)—consistent with hierarchical Bayesian frameworks [15,35].

These findings reveal distinct neural implementations of dynamic expectation parameters in nociceptive processing, proposing a dual-control model: strength amplifies nociceptive salience and cortical integration through hierarchical gain modulation, while precision refines predictive accuracy via cross-stage sensory filtering. This mechanistic segregation advances our understanding of how the brain dynamically balances adaptive updating (strength-driven amplification) with perceptual stability (precision-driven attenuation)—a principle with relevance for understanding both healthy pain regulation and maladaptive predictive processes in chronic pain.

### Expectation-induced modulation of anticipatory brain oscillations

Analysis of anticipatory EEG signals further indicates that expectation strength and precision engage dissociable, frequency-specific mechanisms to shape preparatory neural states. Under heightened expectation strength, we observed suppressed centro-parietal θ-band activity—a pattern interpreted as reduced engagement of posterior conflict-monitoring processes. Whereas midfrontal θ is classically linked to threat detection and cognitive control [45], centro-parietal θ has been associated with prediction–sensory mismatches [46,47]. Strong magnitude expectations may therefore diminish the need for mismatch monitoring, consistent with more efficient allocation of cognitive resources. This θ suppression co-occurred with fronto-central α-desynchronization, replicating prior findings [4,5,48] and reflecting reduced cortical inhibition and enhanced preparatory readiness [49] in anticipation of high-intensity nociceptive input. An alternative, and not mutually exclusive, account is that α-modulation indexes action-oriented preparatory states—such as defensive motor readiness [48,50], possibly supported by the action-mode network [51,52] and the somato-cognitive action network [53].

Expectation precision yielded a complementary spectral profile. Contralateral sensorimotor α-synchronization increased with higher precision, aligning with evidence that primary sensory cortices encode uncertainty through α-band modulation [54–56]. In this context, elevated α power likely suppresses task-irrelevant somatosensory input to enhance the fidelity of anticipated nociceptive signals. This interpretation is consistent with the well-established view that α-oscillations implement adaptive sensory filtering that regulates the flow of behaviorally relevant information [57–60], here expressed in a precision-dependent manner during pain anticipation. Concurrently, precision was also associated with midfrontal β enhancement, a frequency linked both to the maintenance of internal models and to top-down motor preparatory control [61–63], suggesting that confident predictions recruit β-mediated circuits to stabilize belief states while readying regulatory motor systems. These spectral dissociations point to a hierarchical organization of anticipatory processing: expectation strength globally elevates attentional readiness via θ/α desynchronization, whereas expectation precision locally sharpens sensory predictions through focal α/β synchronization. The opposing α patterns—suppression over prefrontal sites to promote vigilance and enhancement over sensorimotor cortex to gate irrelevant input—illustrate how the brain can simultaneously boost global alertness and refine sensory specificity in preparation for anticipated pain.

### Brain α-oscillations as dual oscillatory mechanisms underlying predictive control

Our mediation analyses delineate a frequency- and region-specific architecture through which distinct components of dynamic expectation modulate pain processing via segregated α-oscillatory mechanisms. Specifically, expectation strength elicited anticipatory fronto-central α-desynchronization, reflecting disinhibition of cortical vigilance networks. This effect was mediated by a right-lateralized DLPFC–SM1 circuit, consistent with the DLPFC’s established role in top-down expectation modulation [64–66] and attentional amplification processes [67–69]. Notably, the strength-specific mechanism uniquely recruits mPFC α-oscillations, implicating its self-referential valuation systems in threat salience amplification [70–74]. This mechanism may facilitate aversive learning by enhancing the encoding and subjective appraisal of stimulus–outcome contingencies [75,76]. In contrast, precision-driven suppression was mediated by α-synchronization in contralateral DLPFC and SM1, suggesting a dual gating mechanism: cognitive precision filtering via DLPFC and sensorimotor suppression via SM1. While both expectation components engaged a shared DLPFC–SM1 axis, only strength recruited mPFC α-activity—highlighting its role in threat-related salience processing. In contrast, the absence of mPFC involvement in precision-driven modulation suggests that precision operates via domain-general predictive optimization mechanisms, rather than mechanisms specifically tuned to threat evaluation.

These dual mechanisms reconcile two classical paradoxes of α-oscillations—their roles in both attentional facilitation (via desynchronization) [77,78] and sensory inhibition (via synchronization) [58,79]. We propose that α-rhythms act as a multiplexed cortical control system that—depending on expectation context—either prioritizes stimulus vigilance (strength mechanism) or enforces sensory filtering (precision mechanism). The shared DLPFC-SM1 hub suggests this circuit integrates both expectation dimensions into unified predictive commands, while mPFC’s exclusive link to strength underscores its specialization in modulating threat appraisal.

From a translational perspective, these segregated α-mediated mechanisms identified here may help generate hypotheses for future clinical research, although the present findings are derived entirely from acute, experimentally evoked pain in healthy individuals. Several observations from the chronic pain literature suggest conceptual links worth exploring. Chronic pain conditions are increasingly understood within predictive-processing frameworks that emphasize overweighted priors, impaired belief updating, and dysregulated precision signaling [17,80]. Empirically, many chronic pain populations exhibit maladaptive expectations and disrupted predictive processing [81–85], as well as abnormal α-band oscillatory activity [86–89]. Within this conceptual landscape, our results may offer mechanistic insight: the DLPFC–SM1 α-synchronization identified here appears to support integrative gain control over dynamic expectations, whereas mPFC α-activity selectively tracks expectation strength. These mechanistic distinctions resonate with theoretical accounts proposing that chronic pain involves altered top-down modulation and hypervigilant threat appraisal. While highly speculative at this stage, these findings may motivate future studies examining whether α-rhythm–based neuromodulation or neurofeedback could help restore more adaptive predictive control in chronic pain.

## Conclusions

This study combined dynamic computational modeling with high-temporal-resolution EEG to delineate how evolving expectations modulate pain perception at both behavioral and neural levels. By disentangling two key dimensions of expectation—strength and precision—we identified dissociable anticipatory α-oscillatory mechanisms: expectation strength suppressed fronto-central α power and enhanced pain-evoked responses, whereas precision increased contralateral sensorimotor α-synchronization and dampened nociceptive processing. Source-level analyses further revealed a right-lateralized DLPFC–SM1 circuit integrating both components, with mPFC uniquely recruited by strength-related modulation. These findings position cortical α-rhythms as flexible, frequency-specific channels for predictive control in pain. Critically, the convergence of computational, electrophysiological, and behavioral results—supported by Bayesian inference and pooled mega-analysis—underscores the robustness of these effects. Together, this work advances a mechanistic framework linking dynamic expectations to pain processing, offering theoretical insights into predictive coding and a foundation for future translational research in chronic pain.

## Materials and methods

### Participants

Sample size estimation was conducted using G*Power 3.1.9.7 [90] for a rmANOVA design with four within-participant conditions, yielding a required sample of 62 participants to achieve 0.99 power (α = 0.01, medium effect size *f* = 0.25). Seventy-four healthy, right-handed participants (35 females; mean age: 20.43 ± 0.23 years [mean ± SEM]) were recruited through online advertisements. All had normal or corrected-to-normal vision. Exclusion criteria included current or recurrent pain, neurological or skin disorders, psychiatric conditions, pregnancy or menstruation (for female participants), and regular medication use. Two participants were excluded due to equipment failure or misunderstanding of the procedure, yielding 72 participants for final analysis. All participants provided written informed consent and received financial compensation. The study was approved by the local ethics committee at Shenzhen University (PN-202200071) and conducted in accordance with the Declaration of Helsinki.

### Stimulation and experimental design

Noxious heat stimuli were delivered via an Nd:YAP laser (1,340 nm wavelength, 4 ms pulse, 7 mm beam) to the dorsum of the left hand. To prevent tissue damage and minimize habituation or sensitization [5,8], the stimulation site was slightly shifted after each pulse. Specifically, the experimenter repositioned the laser beam by approximately 1 cm in a random direction within a predefined 4 × 4 cm^2^ target area. This widely used procedure avoids repeated stimulation of the same cutaneous spot while maintaining consistent stimulation geometry across trials. Stimulus intensities for LP (3.25 J, NRS = 44.29 ± 4.28) and HP (3.75 J, NRS = 61.07 ± 4.44) were calibrated in a pilot study (7 participants) using incremental energy levels (2.75–3.75 J) and a NRS ranging from 0 (no pain sensation) to 100 (unbearable pain sensation). These two stimulus intensities ensured clearly perceptual discrimination while keep experiment safety. Only participants who reliably distinguished the intensities continued the formal experiment.

We employed a cued-pain paradigm based on the previous studies [5,8], where two predictive visual cues—HE and LE—were paired with HP and LP stimuli, respectively, with a 75% reinforcement probability. This design included four conditions: LE cues were followed by LP (75%) and HP (25%), while HE cues were followed by LP (25%) and HP (75%). The task schema is shown in Fig 1A (lower panel). Each trial began with a jittered fixation period lasting 2–3 s, followed by a 2-s predictive cue (diamond or square). After a jittered anticipation interval of 4–6 s, a brief laser pain stimulus was delivered to the dorsum of the left hand. Two seconds after pain delivery, participants verbally reported their perceived pain intensity and unpleasantness using the same 0–100 NRS. Trials were pseudorandomized such that no more than three consecutive trials shared the same cue or pain intensity. Invalid trials where the cued and delivered intensities mismatched, were sparsely interleaved, with no more than two presented consecutively. Cue-pain contingencies were counterbalanced across participants.

The experimental protocol comprised four consecutive runs of 40 trials each. Following each run, participants evaluated predictive cues across four dimensions—anticipated pain intensity, unpleasantness, anxiety, and fear—using the same 0–100 NRS (Fig 1A, upper panel). Inter-run intervals of 3 min were implemented to minimize fatigue. To control for potential learning confounds, explicit disclosure of cue-pain contingencies preceded experimental procedures. The main experiment was preceded by two preparatory phases: a 10-stimulus familiarization session exposing participants to varying pain intensities, followed by a 16-trial practice session replicating the experimental paradigm’s cue-pain associations and technical parameters.

### Manipulation check of cue-based expectation

To formally check whether participants’ expectations are successfully manipulated by the cued-pain paradigm, Bayesian paired-sample *t*-tests were conducted on offline ratings for LE and HE cues. These analyses examined whether participants reported higher expected pain intensity and greater negative affect in response to HE cues compared to LE cues. BFs were computed as the ratio of the likelihood of the data under the alternative hypothesis (H₁: a significant difference between HE and LE cues) to the likelihood under the null hypothesis (H₀: no difference between HE and LE cues). A BF > 10 indicated strong evidence in favor of an effect, whereas a BF < 1/10 provided strong evidence supporting its absence [29]. Bayesian rmANOVAs were also conducted on the trial-wise online pain ratings for further confirmation of the cue-pain contingency. Both the Bayesian paired-sample *t*-tes*t*s and rmANOVAs were implemented in the statistical software packages JASP (https://jasp-stats.org/) with its default priors.

### Computational modeling of dynamic expectations

To characterize trial-by-trial expectation dynamics for the HE and LE cues, we implemented a cue-specific leaky integration model. This approach estimates expectations based on a recency-weighted accumulation of past pain experiences, incorporating memory decay to account for forgetting and reduced influence of older trials [14,23,91]. Crucially, expectations were computed separately for each cue type; only pain ratings from previous trials preceded by the same cue contributed to the expectation estimate. This cue-specific formulation preserves context-dependent updating and prevents cross-contamination between cues.

For a given cue (HE or LE), expectation strength at trial *t* was defined as a recency-weighted mean of past pain ratings:


$$\begin{array}{c} E_{t}=\frac{\sum_{k=\text{1}}^{t-\text{1}}w_{k}\times P_{t-k}}{\sum_{k=\text{1}}^{t-\text{1}}w_{k}} \end{array}$$ (1)

where $P_{t-k}$ is the pain rating from *k* trials earlier, and $w_{k}$ is an exponential decay weight:


$$\begin{array}{c} w_{k}=e^{-k/\omega } \end{array}$$ (2)

The decay constant *ω* (set to 8) determines the effective memory window and was chosen based on prior work showing that this value optimally approximates subjective expected-pain ratings in similar paradigms [14,23].

Expectation precision was estimated as the cue-specific variability of past expectation strengths, following the weighted-variance formulation in Pavy and colleagues 2023 [23]:


$$\begin{array}{c} {Var}_{t}=\frac{\sum_{k=\text{1}}^{t-\text{1}}w_{k}{\left(E_{t-k}-\overset{―}{E_{t}}\right)}^{\text{2}}}{\sum_{k=\text{1}}^{t-\text{1}}w_{k}} \end{array}$$ (3a)

$$\begin{array}{c} \overset{―}{E_{t}}=\frac{\sum_{k=\text{1}}^{t-\text{1}}w_{k}{\times E}_{t-k}}{\sum_{k=\text{1}}^{t-\text{1}}w_{k}} \end{array}$$ (3b)

where the $\overset{―}{E_{t}}$ denotes the cue-specific weighted mean. Precision was defined as the inverse of this variance, such that higher values reflected greater certainty. Because the raw variance distribution was skewed (skewness = 3.14), a power transformation (exponent = 0.25) was applied (skewness = 0.38 after transformation). Finally, trial-by-trial expectation strength and precision were normalized to [0, 1] across participants using min-max scaling. This cue-specific modeling procedure was applied consistently across the current dataset, the two datasets from Jepma and colleagues 2018 [3] with trial-wise subjective expectations, and the dataset from Nickel and colleagues 2022 [5].

### Model validation

To evaluate the construct validity and generalizability of the leaky integration model, we implemented a three-step validation procedure. First, we assessed whether model-derived expectation strength corresponded to participants’ subjective beliefs by correlating trial-averaged model estimates with self-reported expected pain ratings collected after each experimental run. Second, we examined external validity by applying the same computational approach to two independent datasets from Jepma and colleagues 2018 [3], where participants provided trial-by-trial expectation ratings, allowing direct comparison between model-derived and subjective expectations. Third, we quantified whether dynamic, trial-level expectation estimates provided a better account of subjective expectations than static cue labels by conducting Bayesian model comparisons. Full statistical procedures are detailed in Text B in S1 File.

To further establish the robustness of the proposed framework, we compared the leaky integration model with alternative computational accounts of expectation updating, including a reinforcement learning-based Rescorla–Wagner (RW) model, a Bayesian Kalman filter model, and a static random model. The RW and Kalman filter models were implemented following established specifications from previous work [3]. All models were fitted to both the current dataset and the independent validation dataset from Jepma and colleagues (2018) [3]. Formal Bayesian model comparison was conducted using the VBA toolbox [31] within a random-effects framework to evaluate relative model evidence across participants. In addition, to examine predictive validity at the behavioral level, we conducted LMM analyses in which subjective expectation ratings were modeled as a function of expectation strength generated by each computational model, with cue type included as a fixed effect and participant as a random effect. Pairwise comparisons between LMMs were then used to quantify how well expectation estimates derived from different computational models accounted for subjective expectation reports.

### Statistical inference framework

To confirm the effects of expectation strength and precision on behavioral responses to laser pain, Bayesian LMMs were applied to pain ratings as a function of expectation strength and precision (both treated as fixed effects). The analysis included data across trials (excluding the first trial for each LE and HE cue) and participants. Stimulus intensity (LP versus HP, coded as 1 and 2, respectively) and the expectation strength × precision interaction was incorporated as fixed effects, while participant was treated as a random effect. The model was specified as follows:


Dependent variables ~ Stimulus intensity+Expectation strength×Precision+(1 |Participant)

For the fixed effects of interest (stimulus intensity, expectation strength, precision, and expectation strength × precision interaction), *Est*, 95% HPD, and *P*p values were extracted for statistical inference. Specifically, compelling evidence supporting the effect is considered if 95% HPD excludes 0 and *P*p exceeds 97.5% [92].

### EEG acquisition and preprocessing

Participants seated in front of a screen to complete the experimental task while their continuous EEG signals were recorded. EEG data were acquired using a 32-channel EEG cap with Ag/AgCl scalp electrodes positioned according to the International 10–20 system (eego mylab, ANT Neuro, Enschede, the Netherlands). The signals were recorded with a band-pass filter of 0.01–100 Hz and sampled at 1,000 Hz. CPz served as the online reference, and AFz as the ground electrode. Prior to the experiment, electrode impedances were maintained below 10 kΩ.

EEG data were preprocessed using the EEGLAB toolbox [93] and custom MATLAB scripts. A 50-Hz notch filter was applied to suppress line noise, followed by band-pass filtering between 1 and 100 Hz. Data were then re-referenced to the linked mastoid electrodes. For anticipatory oscillation analysis, 4,000-ms epochs were extracted (from −4,000 ms to pain onset). For pain-evoked neural response analysis, 1,500-ms epochs were extracted (−500 ms to +1,000 ms relative to pain onset). Baseline correction was performed using the mean amplitude of the pre-stimulus interval (4,000 ms for anticipatory analysis and 500 ms for pain-related responses). Independent component analysis [94] was recruited to isolate canonical noises including muscular and ocular artifacts.

### Analysis on pain evoked neural responses

To quantify the amplitudes of laser-evoked N2 and P2 components, a 30 Hz low-pass filter was applied to artifact-corrected EEG epochs. The laser-evoked response amplitudes were measured individually for each participant following these steps: (1) A grand-averaged waveform was generated across all trials and conditions at the Cz electrode. (2) Peak and trough detection was performed within the 180–300 ms and 250–500 ms windows to identify the latencies of the N2 and P2 components [95]. (3) Trial-wise amplitudes of N2 and P2 were calculated by averaging a 30-ms window centered on the individual latencies identified in the previous step at Cz [5].

To assess laser-induced oscillatory power, time–frequency decomposition was performed using a windowed Fourier transform with a fixed 250-ms Hanning window [95]. This analysis was conducted over a time range of −500 to 1,000 ms (10-ms intervals) and a frequency range of 1–100 Hz (1-Hz intervals). Spectrograms were baseline-corrected using the pre-stimulus interval (−400 to −100 ms) by subtracting the mean power of this interval from each time-frequency point. For each participant and trial, laser-evoked oscillatory features were extracted by averaging power within predefined time-frequency windows where the respective oscillations reached their maximal response. Building on previous studies investigating pain-related neural oscillation [5,95,96], three key oscillatory features were selected and measured: (1) LEP at 1–10 Hz, 100–400 ms and FC1, FC2, and Cz electrodes; (2) α-ERD at 7–13 Hz, 500–900 ms and P3, Pz, and P4 electrodes; (3) γ-ERS at 60–90 Hz, 200–350 ms, and FC1, FC2, and Cz electrodes.

After extracting trial-by-trial neural responses to laser stimulation, Bayesian LMMs were applied to examine the effects of expectation strength and precision on pain-induced neural activity. The statistical approach mirrored that used for behavioral data analysis.

### Analysis on sensor-level anticipatory oscillations before pain delivery

Power spectral analysis of EEG data during the pain anticipation phase was conducted using the in-house FieldTrip function *ft_freqanalysis* [97] with a multitaper frequency transform. Non-overlapping 4-s pre-stimulus epochs were analyzed to extract absolute power spectral density cross 1–100 Hz with a resolution of 0.25 Hz (1/epoch length in seconds). To minimize spectral leakage, discrete prolate spheroidal sequence tapers were applied before Fourier transformation. Channel-specific power spectra were computed for each epoch using Fast Fourier Transform (FFT). For subsequent analyses, mean power was calculated for each channel within the following frequency bands: θ (4–7 Hz), α (7–13 Hz), β (13–30 Hz), and γ (30–90 Hz). To identify where anticipatory oscillations were modulated by expectation strength and precision, we used a deliberate two-stage statistical pipeline designed to separate spatial discovery from quantitative inference. First, a whole-scalp, channel-by-channel LMM was implemented using custom MATLAB scripts:


Oscillatory power ~ Expectation strength×Precision+(1 |Participant))

This exploratory step provides high sensitivity for detecting potential spatial patterns while preserving within-participant variance. Because this procedure involves multiple electrodes and frequency bands, *p*-values were corrected using FDR correction [98]. Only spatially coherent clusters (>2 contiguous significant channels) were retained for subsequent analysis. This stage served only to localize candidate electrodes and did not contribute to inferential claims. Then, for each effect, oscillatory power was averaged across the identified electrode cluster, and Bayesian LMMs were applied to quantify posterior probabilities and 95% HPD intervals. All inferential conclusions regarding expectation strength, precision, and their interaction were therefore derived from Bayesian statistics. This frequentist–Bayesian approach balances computational feasibility during high-dimensional spatial searches with the rigorous probabilistic inference afforded by Bayesian models.

### Source reconstruction

To localize cortical sources of expectation-modulated α-oscillations, we performed source reconstruction using linearly constrained minimum variance (LCMV) beamformers [99] in FieldTrip [97]. The leadfield matrix was computed for a three-dimensional grid (1-cm resolution) based on a three-shell boundary-element volume conduction model (Montreal Neurological Institute template), with dipole orientations constrained perpendicular to the cortical surface. Sensor-level signals were bandpass-filtered (7–13 Hz), and the data covariance matrix was estimated from pre-stimulus intervals (−4 to 0 s) across all trials. LCMV spatial filters were optimized for α-band analysis with a central frequency of 10 Hz and spectral smoothing of 3 Hz, applying 5% regularization and fixed dipole orientations following established protocols [8].

Source-level α power estimates were anatomically parcellated using the Automated Anatomical Labeling (AAL) atlas [100]. We defined 12 ROIs (Fig 8A): mPFC and SPL, as well as bilateral SM1, DLPFC, IFG, IPL, and TL. Grid points within each AAL-derived ROI (see Text F and Table D in S1 File) were averaged to obtain regional power values. These coarse, theory-driven ROIs align with large-scale networks implicated in predictive processing and pain modulation [101,102] and are appropriate given the spatial resolution of a 32-channel montage, providing stable summary estimates without implying fine-grained anatomical precision. To further evaluate the robustness of LCMV reconstruction at this electrode density, we applied the identical source-analysis pipeline to a 32-channel subset of the independent 64-channel dataset from Nickel and colleagues, 2022 [5]. Source-level α power derived from the reduced and full montages showed high concordance across ROIs (*rs* ≥ 0.91; *Est*s ≥ 0.76; see Text F and Fig E in S1 File), indicating that the spatial patterns relevant to α-oscillatory mechanisms are well preserved at 32 channels. Consistently, scalp topographies of anticipatory α activity were also highly similar across the two studies (*r* = 0.93), supporting the reliability of α-based spatial inferences across montage densities.

Trial-by-trial effects of expectation strength and precision on ROI-level α power were then assessed using LMMs, followed by Bayesian LMMs to obtain full posterior estimates for inferential conclusions.

### Multilevel mediation analysis

To examine how the brain encodes expectation strength and precision during pain anticipation and integrates these signals into pain perception, we conducted multilevel mediation analyses using trial-wise data. Expectation strength and precision were treated as independent variables, sensor- and source-level anticipatory oscillations as mediators, and pain-evoked responses as outcome variables. Analyses were performed using the Multilevel Mediation toolbox (https://github.com/canlab/MediationToolbox). The significance of all effects was assessed via a bootstrap procedure with 10,000 resamples, and all tests were two-tailed. Given the emphasis on identifying significant mediation effects of anticipatory oscillations, FDR corrections [98] were applied to control for multiple comparisons.

### Mega-analysis incorporating the external dataset from Nickel and colleagues [5]

To assess the robustness and generalizability of our findings, we conducted an individual-participant-level mega-analysis combining the present dataset with the external dataset reported by Nickel and colleagues [5]. Both datasets were reprocessed using an identical preprocessing and analytic pipeline to ensure methodological harmonization. Following this, the two datasets were pooled at the participant level, and Bayesian LMMs were applied to anticipatory oscillations and pain-evoked responses, with dataset origin included as a covariate. The Bayesian LMMs were specified as *DV ~ Stimulus intensity + Expectation strength × Precision + Study + (1 | Participant)* for pain-evoked responses and *DV ~ Expectation strength × Precision + Study + (1 | Participant)* for anticipatory pre-stimulus activities. The mediation analyses followed the same multilevel framework as in the main analyses.

## Supporting information

S1 File Supplementary methods, supplementary results and corresponding tables and figures are included in this file. (PDF)

## Abbreviations

BFs: Bayes factors
HE: high-expectation
HP: high-intensity pain
HPD: highest probability density
IFG: inferior frontal gyrus
LE: low-expectation
LEP: laser-evoked potential
LMMs: linear mixed-effects models
LP: low-intensity pain
mPFC: medial prefrontal cortex
rmANOVAs: repeated-measures ANOVAs
RW: Rescorla–Wagner
α-ERD: α-event-related desynchronization
γ-ERS: γ-event-related synchronization
