# Supplementary material for: Dynamic expectation strength and precision shape human pain perception through shared and dissociable α-oscillatory mechanisms
Source: PLoS Biol. 2026 Mar 2;24(3):e3003675. doi: 10.1371/journal.pbio.3003675 (PMC12965688; doi:10.1371/journal.pbio.3003675)
Supplement: S1 File — (PDF) [file pbio.3003675.s001.pdf]

Supporting Information for

Dynamic Expectation Strength and Precision Shape Human Pain Perception through Shared and Dissociable α-Oscillatory Mechanisms

Jia Li, Shihao Chen, Libo Zhang, Lingling Weng, Xinxin Lin, Yiheng Tu, Weiwei Peng

Corresponding author:

E-mail: ww.peng0923@gmail.com (WP)

Supporting Information

The supplementary materials provide additional analyses and methodological details supporting the main findings.

- **Text A** presents replication results evaluating how static expectations (i.e., predictive cues) influence pain perception. Specifically, Text A1 demonstrates the Bayesian repeated-measures analysis of variance (rmANOVAs) results (summarized in Table A), and Text A2 presents Bayesian linear mixed-effects model (LMM) comparisons (visualized in Fig A).
- **Text B** validates the construct validity and external validity of the leaky integration model. Text B1 assesses the correspondence between the model-derived expectation strength and the self-reported block-wise expectation ratings. Text B2 examines the external validity applying the same computational approach to independent datasets with trial-by-trial expectation ratings. B3 presents the direct comparison of dynamic expectation and static, categorical expectation in explaining actual expectation.
- **Text C** presents the formal model comparisons among the leaky integration model and other prediction-error-driven models (Rescorla-Wagner and Bayesian model), and a random static model (see Table B and Figs B−C).
- **Text D** illustrates the replication of our main results when controlling the pain habituation and cue validity which could confound the expectation effects. Text D1 yields that the expectation effects on behavioral ratings remained robust when pain repetition was included as covariate. Text D2 suggests that the expectation effects persist when explicitly controlled the cue validity (see Table C).
- **Text E** illustrates the exploratory analyses of the expectation modulations on anticipatory EEG responses based on time-frequency decompositions (see Fig D).
- **Text F** presents the methodological details of our source-level framework, covering both brain region parcellation and the validation of source reconstruction across different electrode montages. Specifically, Text F1 details the correspondence between Automated Anatomical Labeling (AAL) atlas regions and our predefined regions of interest (ROIs), clarifying the

source-level analysis framework (see Table D). Text F2 depicts the well convergence between 32-channel and 64-channel montages in source reconstruction (see Fig E).

- **Text G** describes the mega-analyses regarding behavioral pain ratings (Text G1 and Fig F), time-domain LEP responses (Text G2 and Fig G), pain-induced neural oscillations (Text G3 and Fig H), and pre-stimulus anticipatory EEG oscillations (Text G4 and Fig I). The mega-analyses serve as robustness check for the observed expectation modulations on pain perception.

Text A. Static expectation effects on pain

A1. Replication using Bayesian repeated measures ANOVA. Bayesian rmANOVAs were systematically applied to condition-averaged datasets to quantify evidence for three principal experimental factors: Stimulus Intensity (nociceptive input), Expectation (top-down modulation), and their interaction term representing Prediction Errors (PEs = Stimulus Intensity × Expectation). Bayesian factor (BF) quantification for all model components across dependent variables is tabulated in Table A.

Decisive evidence emerged for dual determinants of subjective pain reports: Nociceptive input demonstrated extreme evidential support (Pain intensity: BF = 4.62×10^{14}; Unpleasantness: BF = 1.92×10^{16}) alongside substantial expectation modulation (Pain intensity: BF = 2.76×10^{11}; Unpleasantness: BF = 482.25). This pattern manifested behaviorally as elevated ratings for high pain (HP) versus low pain (LP) trials, and for high expectation (HE) versus low expectation (LE) cues. Crucially, the interaction term failed to reach evidential thresholds (Pain intensity: BF = 0.87; Unpleasantness: BF = 2.42), indicating independent rather than synergistic contributions of bottom-up sensory and top-down cognitive processes.

Spectral analysis of neural oscillations demonstrated robust intensity-dependent modulation across multiple frequency bands. Gamma-band event-related synchronization (γ-ERS) exhibited significant enhancement following HP stimuli compared to LP trials (BF = 24.28), paralleled by intensity-specific potentiation of LEP responses (N2: BF = 5.91×10^{15}; P2: BF = 1.66×10^{17}; LEP: BF = 2.27×10^{12}). Notably, alpha-band event-related desynchronization (α-ERD) showed divergent patterns, with Bayesian evidence rejecting intensity effects (BF = 0.57). Further analysis revealed null support for expectation-driven modulations across all neurophysiological metrics (BF ≤ 0.13), with interaction terms similarly failing to reach evidential thresholds (BF ≤ 0.05). These spectral dynamics align with event-related potential findings, collectively establishing that oscillatory signatures of nociceptive processing—whether in time-domain evoked responses or time-

frequency representations—predominantly reflect bottom-up sensory encoding rather than top-down predictive signals.

Table A. Bayesian factors (BFs) for main and interaction effects

DV	Stimulus intensity	Expectation	Interaction
Pain intensity	**4.62×10^{14}**	**2.76×10^{11}**	0.87
Unpleasantness	**1.92×10^{16}**	**482.25**	2.42
N2 amplitude	**5.91×10^{15}**	0.38	0.90
P2 amplitude	**1.66×10^{17}**	0.16	0.24
LEP	**2.27×10^{12}**	0.48	0.29
α-ERD	0.57	0.13	0.05
γ-ERS	**24.28**	0.36	0.35

Note. DV: dependent variable. Compelling evidence in favor of the effect of interest (BF > 3) is marked bolded. Data supporting this table are available at https://doi.org/10.5281/zenodo.18503056.

A2. Bayesian model comparisons. Complementing condition-level analyses, we implemented trial-wise Bayesian linear mixed model (LMM) comparisons to quantify predictive contributions of:

1. Nociceptive input (stimulus intensity: HP = 1 vs LP = 0)
2. Cognitive expectation (prior probability: LE = 0.25 vs HE = 0.75)
3. Prediction error (PE = stimulus intensity - expectation, rectified for aversive coding [1])

Three competing LMMs were evaluated:

- *Sensory-driven model*: Single-predictor intensity effects (INT)
- *Dual-process model*: Combined intensity + expectation (INT + EXP)
- *Predictive coding model*: Expectation + PE interactions (EXP + PE)

Stimulus-expectation interactions followed established aversive PE formulations [1], preserving positive discrepancies (unexpected pain) while truncating negative values. Full mathematical specification and theoretical justification align with validated neurocomputational frameworks (see Nickel et al 2022 [1] for derivations). Bayesian model evidence was computed via BayesFactor

package (version 0.9.12-4.7, [2]), with log-transformed Bayes factors log(BF) quantifying relative predictive superiority. Positive log(BF) values indicate evidence favoring row models over column models in paired comparisons.

Results of Bayesian model comparisons are depicted in Fig A. Conscious pain perception demonstrated distinct computational mechanisms from neurophysiological encoding. For subjective pain reports, dual-process models integrating both nociceptive input and cognitive expectation substantially outperformed sensory-driven models, yielding decisive evidence magnitudes (pain intensity: log(BF) = 71.18; unpleasantness: log(BF) = 26.02). Strikingly, predictive coding models incorporating expectation-prediction error dynamics catastrophically failed to explain behavioral responses, exhibiting extreme negative evidence values (pain intensity: log(BF) = –280.59; unpleasantness: log(BF) = –149.18). Contrastingly, neural response patterns revealed unimodal sensory dominance. Sensory-driven models optimally accounted for laser-evoked potential components (N2/P2 amplitudes) and oscillatory dynamics (γ-ERS/α-ERD), showing consistent evidence superiority over dual-process models (log(BF) ≤ –0.69) and predictive coding models (log(BF) = –3.11). Crucially, neither expectation nor prediction error terms provided explanatory value for any neural metric, with all comparative models yielding negative evidence (log(BF) < 1).

These findings fully replicated the results in Nickel et al 2022 [1]: pain ratings result from the integrated effects of bottom-up sensory input and top-down expectation, while most EEG responses to noxious laser pain were primarily driven by the bottom-up sensory input.

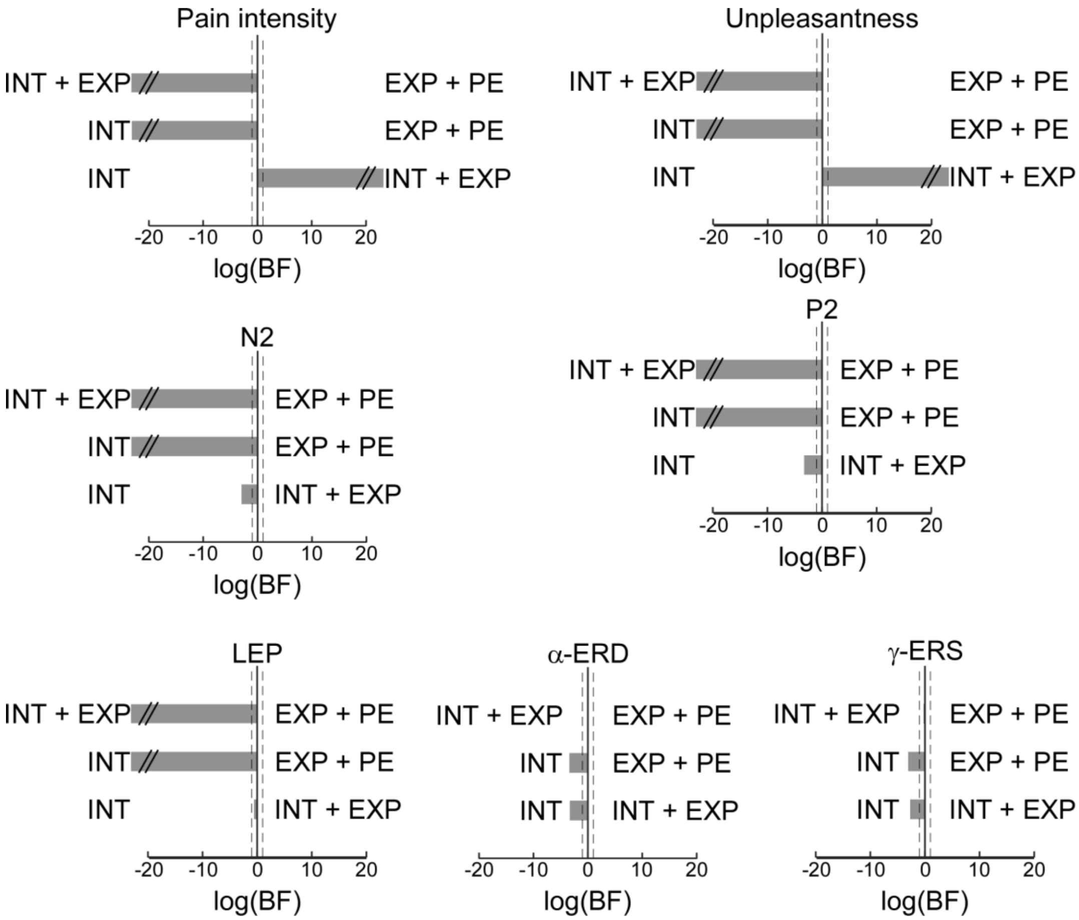

Fig A. Bayesian model evidence comparisons.
Pairwise model comparisons evaluate three computational architectures: INT model, INT + EXP model, and EXP + PE model. Bar heights represent natural-logarithm-transformed Bayes factors log(BF), with truncated bars (||) indicating extreme evidence magnitudes (|log(BF)| > 20). Vertical dashed lines demarcate thresholds of strong evidence: log (BF) = –1 and log (BF) = 1. INT: Dependent variable ~ stimulus intensity + (1|participant); INT + EXP: Dependent variable ~ stimulus intensity + expectation + (1|participant); EXP + PE: Dependent variable ~ expectation + PE + (1|participant). Data supporting this figure are available at https://doi.org/10.5281/zenodo.18503056.

Text B Model validation

A three-step validation procedure is implemented to systematically evaluate the construct validity and external validity of the leaky integration model.

B1. Construct validity. To examine the construct validity of the leaky integration model, we firstly compared the differences of modeled expectations between the informative cues through Bayesian LMMs. The LMMs are specified with the following syntax: *Modeled expectation strength/precision ~ Cue type + (1* | *Participant)*. These analyses yielded robust evidence in favor of the higher modeled expectation strength (*Est* = 14.49, 95% HPD = [14.15, 14.81], *P*p = 1.00) and lower precision (*Est* = –0.02, 95% HPD = [–0.03, –0.02], *P*p= 1.00) following HE compared to LE cues.

Besides, for each cue type, we directly computed the between-subject Pearson correlations between averaged modeled strength and subjective ratings after each experiment run. High correlations were yielded for both the LE ($r = 0.67$, $p < 0.001$) and HE ($r = 0.85$, $p < 0.001$) cues. These results together support the construct validity of the leaky integration model demonstrating the high fidelity of the computational estimates.

B2. External validity. The leaky integration model was also implemented on the two independent datasets reported by Jepma et al 2018 [3] in which participants reported their trial-wise pain expectation after each cue. We thus implemented Bayesian LMMs to estimate the within-participants correlation between the modeled expectation strength and self-reported ratings. For each study and each cue type, the LMM model was specified as: *Subjective expectation ~ Modeled expectation strength + (Modeled expectation strength* | *Participant)*. Specifically, both random intercept and random slopes were included to account for the different participants. As supplementary, between-participants correlations were also calculated by averaging the modeled and subjective expectations for each study and each cue. Both the robust within-participant correlation and high between-participant correlation confirm the external validity of the leaky integration model.

B3. Comparisons of dynamic versus static expectations. To determine whether dynamic expectations outperform static cue labels in explaining subjective expectation strength, we constructed LMMs predicting subjective expectation using either model-derived strength [*Offline rating ~ Modeled expectation strength + (1* | *Participant)*] or cue type [LE/HE; *Offline rating ~ Cue type + (1* | *Participant)*]. Model comparisons were achieved via Bayes factor analysis as described in S1b. Decisive evidence was yielded in favor of the dynamic expectations in explaining actual pain expectations (Dynamic > Static: BF = 1.17×10^{17}).

Similar model comparison is also conducted on the independent datasets form Jepma et al 2018 [3]. In this case, trial-by-trial subjective expectation and modeled expectation strength were treated as dependent and independent variables respectively. Specifically, we combined the two datasets while controlling the potential confound of stimulation site by including it as covariate. The models were defined as *Subjective expectation ~ Modeled expectation strength/Cue type + Stimulation site + (1* | *Participant)*. Again, dynamic expectations outperform the static cue labels in explaining actual pain expectations (Dynamic > Static: BF = 7.03×10^{16}).

Text C. Leaky integration model outperforms prediction error models

We implemented formal model comparisons between the current leaky integration model and prediction-error models including the Rescorla–Wagner delta-rule (RW) model and a Bayesian Kalman filter model. These analyses were conducted on both the current dataset and the independent dataset from the study 1 of Jepma et al 2018 [3].

C1. Model specifications. Both the RW model and Bayesian model strictly follow the specifications in Jepma et al 2018 [3] and the confirmation bias was also modeled. A random model was also tested to ensure that any advantage of the leaky model could not be explained by trivial mean-level estimation.

Leaky integration model. The algorithms of leaky integration model were fully described in the main text.

RW model. Pain perception for a given trial P_t was calculated as the weighted sum of expectation E_t and the noxious input N_t:

$$P_t = \gamma E_t + (1 - \gamma) P_t \tag{1}$$

where the γ controls the relative influence of expectation versus noxious stimulation when shaping pain perceptions. The expectation evolves based on the prediction error δ_t determined by the learning rate α:

$$E_t = E_{t-1} + \alpha \delta_t \tag{2a}$$

$$\delta_t = P_t - E_t \tag{2b}$$

Specifically, we varied the learning rate according to whether the sign of δ_t is consistent with the initial belief associated with the cue (c):

$$\alpha_t = \begin{cases} \alpha_c, & if\ (\delta_t > 0\ and\ c = high)\ or\ (\delta_t < 0\ and\ c = low) \\ \alpha_i, & if\ (\delta_t < 0\ and\ c = high)\ or\ (\delta_t > 0\ and\ c = low) \end{cases} \tag{3}$$

Bayesian Kalman filter model. The model assumes pain perception P_t as precision-weighted integration between the prior expectation E_t and the sensory evidence N_t:

$$P_t = \frac{\sigma_\varepsilon E_t + (\sigma_\psi + V_t) N_t}{(\sigma_\varepsilon + \sigma_\psi + V_t)}, \qquad with \ \sigma_\varepsilon = 1 \tag{4}$$

The expectation update follows the Kalman filter fashion which assumes the objective cue-outcome associations vary over time according to the Gaussian random walk. Full descriptions of the Bayesian Kalman filter refer to Jepma et al 2018 [3].

Random static model. In the random model, the pain expectation at given trial strictly follow the initial belief according the cue (μ_{low} for low cue and μ_{high} for high cue) with global reporting bias (b), and are not evolved throughout the experiment:

$$E_t = \begin{cases} \mu_{low} + b, & if \ c = low \\ \mu_{high} + b, & if \ c = high \end{cases} \tag{5}$$

C2. Results of the Bayesian model comparisons. All four models were fitted both to the publicly available dataset from Study 1 of Jepma et al 2018 [3], hereafter validation dataset, and to the current dataset. In the validation dataset, the structured models (leaky, RW, Bayesian) produced highly similar trial-wise expectation trajectories, whereas the random model failed to capture the data. Bayesian model selection was achieved using the VBA toolbox [4] through random effect analysis. This comparison yields the model frequency (the proportion of participants best explained by each model), the model exceedance probability (the probability that a given model is more frequently used than all others in the set), and the protected exceedance probability (the exceedance probability corrected for chance-level observations). These values are correlated and would be considered together when selecting the best fit model. Results showed that the leaky model outperforms all other models (Fig B) demonstrating highest model frequency and protected exceedance probability (PEP = 0.945). In the current dataset (Fig B, despite having only block-wise expectation ratings, the leaky model again showed the highest model frequency and a PEP = 1.000, with considerably weaker evidence for the RW and Bayesian models.

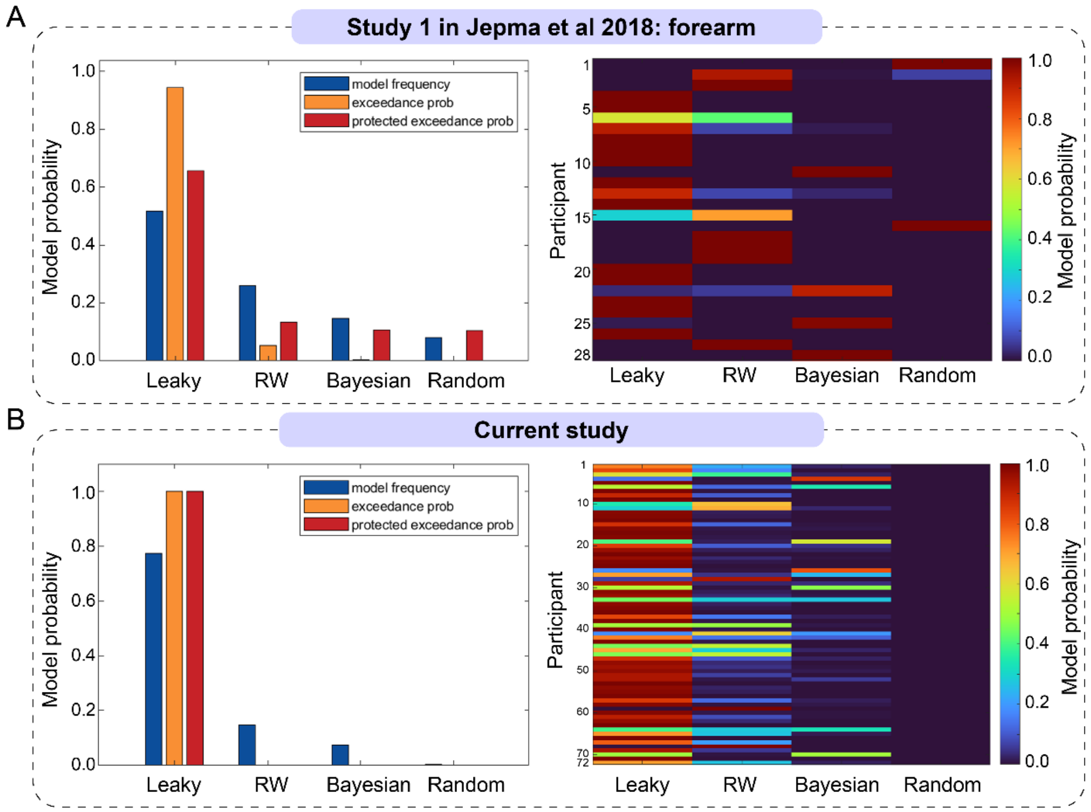

Fig B. Bayesian model comparison results.
Formal Bayesian model comparison results for (A) the validation dataset from Jepma et al 2018 [3] and (B) the current dataset. Left panels show group-level Bayesian model selection based on model evidence, including model frequency, exceedance probability, and protected exceedance probability. Right panels display participant-level model probabilities (each row represents one participant; color scale reflects the posterior probability from 0 to 1), illustrating which model best explained individual expectation reports.

C3. Predictive validity among the candidate computational models. To quantify the predictive validity, we conducted formal pairwise model comparisons to determine which model-derived expectation strength best describe the subjective expectation reported by participants. Specifically, we specified four LMM models where subjective expectations (trial-wise subjective expectation in the validation dataset, and the offline expectations after each task run in the current dataset) were treated as dependent variables, and the model-derived expectation strengths (RW model, Bayesian Kalman filter, Leaky integration, and Random model) and the cue type as fixed factors. The LMM model syntax was as follow: *Subjective expectation ~ Modeled expectation strength + Cue type + (1 | Participant)*. Pairwise model comparisons are listed in Table B and Fig C. In the validation dataset of Jepma et al [3], we found extreme evidence that model expectation strength from the Leaky integration model better describe the subjective expectation than that from RW

model (BF = 2.705 × 10^{49}) and Bayesian Kalman filter (BF = 2.705 × 10^{19}). Similar results were also yielded in the current dataset (Leaky integration vs. RW: BF = 118.353; Leaky integration vs. Bayesian Kalman filter: BF = 5.027). These model comparisons again support the validity of the Leaky integration model in describing the real expectation strength.

Table B. loge[Bayes factors] for model comparisons

		Leaky	RW	Bayesian	Random
Jepma et al 2018	Leaky	0	–	–	–
	RW	113.822	0	–	–
	Bayesian	44.668	−69.154	0	–
	Random	450.459	336.637	405.792	0
Current dataset	Leaky	0	–	–	–
	RW	4.774	0	–	–
	Bayesian	1.615	−3.170	0	–
	Random	64.119	59.346	62.504	0

Note. We log-transformed the Bayes factors (BFs) such that a value of 0 that the data are equally likely to occur under both models. Positive values indicate evidence in favor of the model in the row over the model in the column.

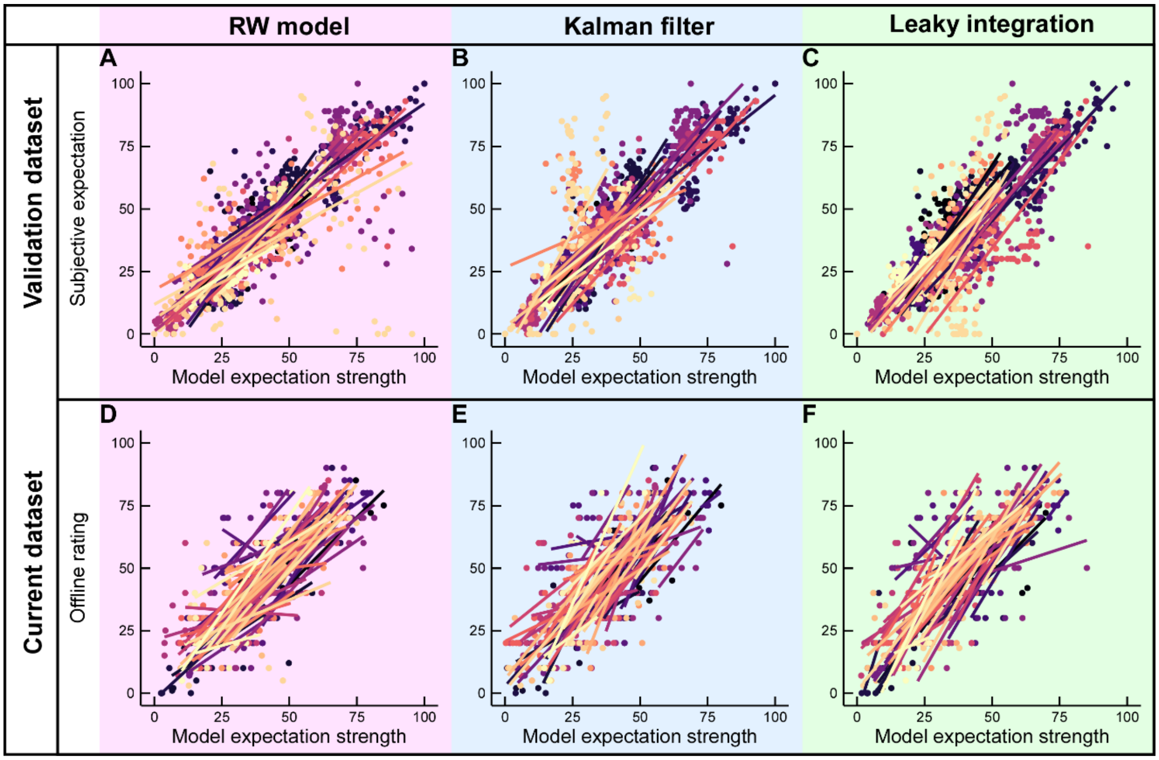

Fig C. Correlations between the model-derived expectation strength and subjective expectations.
(A−C) Participant-level fits among the model expectation from RW model (A), Bayesian Kalman filter (B), and Leaky integration (C), and the subjective expectation in the validation dataset from Jepma et al 2018 [3]. (D−F) Participant-level fits among the model expectation from RW model (D), Kalman filter (E), and Leaky integration (F), and the offline expectation after each task run in the current dataset. Colored lines and dots represent the linear fits and individual data for each participant.

Text D. Expectation effects remained robust after controlling habituation and cue validity

D1. Habituation was included as covariate. To directly test whether habituation might distort expectation estimates, we re-fit the behavioral LMMs with pain repetition included as a covariate. The model syntax was specified as: *Pain intensity ~ Stimulus intensity + Expectation strength × Precision + Pain repetition + (1 | Participant)*. A mild habituation effect was present (*Est* = −0.09, 95% HPD = [−0.11, −0.08], *P*p = 1.00), indicating gradual desensitization. However, expectation strength (*Est* = 23.86, 95% HPD = [21.58, 26.11], *P*p = 1.00) and precision (*Est* = 6.55, 95% HPD = [4.04, 9.04], *P*p = 1.00) remained strong and highly reliable predictors. This demonstrates that expectation effects persist even when habituation is explicitly accounted for, ruling out the possibility that the model simply tracks slow temporal drift.

D2. Cue validity was explicitly controlled. To further reduce the residual correlation between expectation and stimulus intensity, we added cue validity (valid vs. invalid trials) as an additional covariate, since invalid trials partially dissociate the cue from the delivered intensity (LEHP; HELP). When both factors—stimulus intensity and cue validity—were included in the models, expectation strength and precision remained highly robust predictors of both behavioral and neural outcomes (Table C). This demonstrates that the expectation effects cannot be attributed to cue-intensity confounding and instead reflect genuine predictive influences on pain processing

Table C. Bayesian linear mixed-model (LMM) results for pain-related responses after jointly controlling for stimulus intensity and cue validity

DV	Stimulus intensity			Expectation			Precision			Interaction		
	Est	95% HPD	*P*p	Est	95% HPD	*P*p	Est	95% HPD	*P*p	Est	95% HPD	*P*p
Intensity	**18.520**	**[17.884, 19.161]**	**1.000**	**26.863**	**[24.631, 29.102]**	**1.000**	**2.664**	**[0.248, 5.088]**	**0.984**	**–9.501**	**[–14.026, –4.965]**	**1.000**
Unpleasantness	**14.736**	**[14.119, 15.349]**	**1.000**	**20.524**	**[18.326, 22.691]**	**1.000**	**3.496**	**[1.089, 5.857]**	**0.998**	**–8.484**	**[–12.939, –4.044]**	**1.000**
N2 amplitude	**–7.732**	**[–8.255, –7.211]**	**1.000**	**–10.720**	**[–12.554, –8.799]**	**1.000**	**3.138**	**[1.103, 5.194]**	**0.999**	2.363	[–1.605, 6.228]	0.882
P2 amplitude	**6.474**	**[5.970, 6.999]**	**1.000**	**7.971**	**[6.172, 9.777]**	**1.000**	–1.840	[–3.835, 0.133]	0.964	–2.864	[–6.606, 0.907]	0.929
LEP oscillation	**1.678**	**[1.501, 1.853]**	**1.000**	**3.470**	**[2.843, 4.098]**	**1.000**	**–1.513**	**[–2.201, –0.820]**	**1.000**	–0.313	[–1.615, 0.979]	0.683
alpha-ERD	**–0.237**	**[–0.382, –0.093]**	**1.000**	–0.087	[–0.605, 0.428]	0.627	–0.216	[–0.773, 0.329]	0.777	0.480	[–0.575, 1.556]	0.808
gamma-ERS	**0.017**	**[0.007, 0.026]**	**1.000**	**0.053**	**[0.019, 0.087]**	**0.999**	0.003	[–0.033, 0.040]	0.572	–0.011	[–0.082, 0.059]	0.626

Note. All models followed the specification: DV ~ Stimulus intensity + Expectation strength × Precision + Cue validity + (1 | Participant). An effect was considered robust when its posterior probability (*P*p) exceeded 97.5% and its 95% highest posterior density (HPD) interval excluded zero. Robust effects meeting both criteria are indicated in bold.

Text E. Exploratory time-frequency analyses on anticipatory EEG oscillations

We performed exploratory time-frequency decomposition (TFD) to determine the temporal and spectral specificity of expectation strength and precision. We used a windowed Fourier transform with a 250‑ms Hanning window over a frequency range of 1 to 100 Hz (1-Hz intervals) and a time range of −4000 to 0 ms (pain onset), and ran LMMs across time–frequency points within two predefined areas (frontal: Fz, FC1, FC2; contralateral: FC6, C4, T8). These analyses revealed temporally specific modulations broadly consistent with our averaged findings (Fig D). Expectation strength was associated with reductions of theta-to-alpha power around −4000 to −2000 ms before pain onset, reflecting the cortical vigilance. Expectation precision was associated with enhancement of alpha-to-beta activity immediately prior to the pain onset, reflecting attentional filtering and sensory gating. Importantly, these analyses were exploratory: they were restricted to specified areas and did not include correction for multiple comparisons, so their inferential status is tentative.

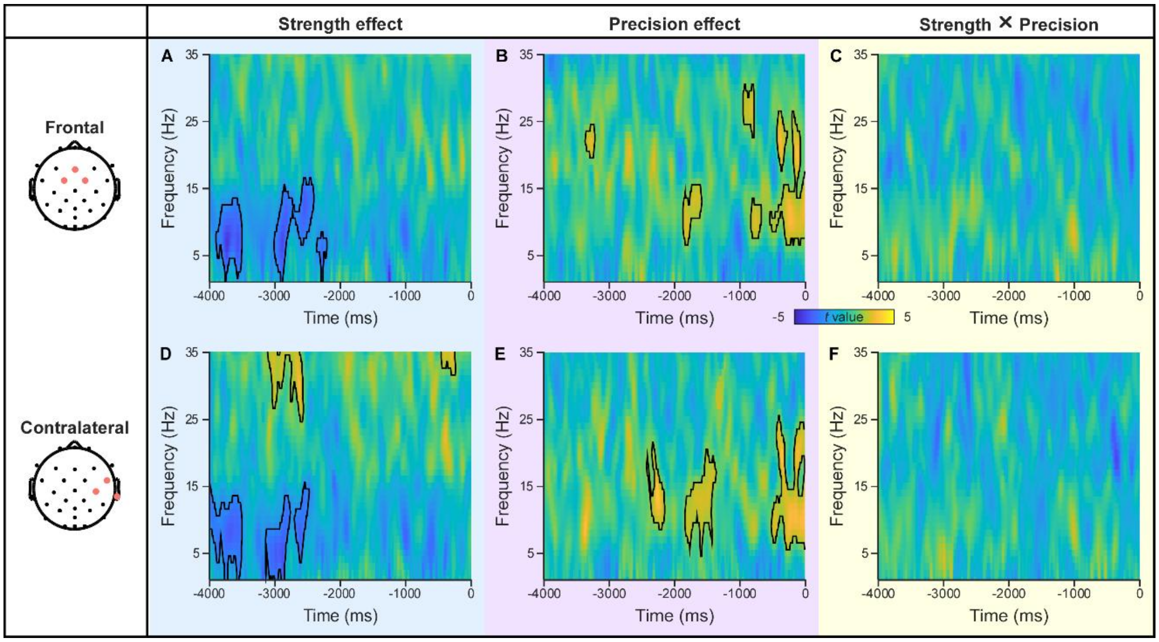

Fig D. Statistics for the LMM on time-frequency representations (TFRs) before pain stimulus.
(A−C) T-statistic maps display the linkages between expectation profiles and TFR at frontal area (Fz, FC1, & FC2). (D−F) T-statistic maps display the linkages between expectation profiles and TFR at electrodes contralateral to pain site (FC6, C4, & T8). Significant clusters (unthresholded, $p < 0.05$) with cluster size lager than 40 and lasting over 150 ms are circled by the white contour line.

Text F. Source-level ROI definition and cross-montage robustness

F1. Definitions of source-level ROIs. Cortical parcellation was implemented through anatomical alignment with the Automated Anatomical Labeling (AAL) atlas [5]. The leadfield matrix elements were anatomically grouped using AAL-defined regions, with subsequent formation of 12 composite ROIs encompassing major cortical structures through systematic aggregation of functionally related AAL subregions. The resultant neuroanatomical mapping scheme is comprehensively presented in Table D.

Table D. Neuroanatomical mapping between composite ROIs and constituent AAL labels

ROI	AAL_Label
lSM1	Precentral_L, Postcentral_L
rSM1	Precentral_R, Postcentral_R
mPFC	Frontal_Sup_Medial_L, Frontal_Sup_Medial_R, Frontal_Med_Orb_L, Frontal_Med_Orb_R, Rectus_L, Rectus_R
lDLPFC	Frontal_Sup_L, Frontal_Mid_L
rDLPFC	Frontal_Sup_R, Frontal_Mid_R,
lIFG	Frontal_Inf_Tri_L, Frontal_Inf_Oper_L, Frontal_Inf_Orb_L
rIFG	Frontal_Inf_Tri_R, Frontal_Inf_Oper_R, Frontal_Inf_Orb_R
SPL	Parietal_Sup_L, Parietal_Sup_R, Precuneus_L, Precuneus_R
lIPL	Parietal_Inf_L, SupraMarginal_L, Angular_L
rIPL	Parietal_Inf_R, SupraMarginal_R, Angular_R
lTL	Heschl_L, Temporal_Sup_L, Temporal_Pole_Sup_L, Temporal_Mid_L, Temporal_Pole_Mid_L, Temporal_Inf_L
rTL	Heschl_R, Temporal_Sup_R, Temporal_Pole_Sup_R, Temporal_Mid_R, Temporal_Pole_Mid_R, Temporal_Inf_R

Note. ROI – region of interest; mPFC – medial prefrontal cortex; DLPFC – dorsolateral prefrontal cortex; SM1 – primary sensorimotor cortex; IFG – inferior frontal gyrus; TL – temporal lobe; IPL – inferior parietal lobule; SPL – superior parietal lobule; l – left; r – right.

F2. Convergence between 32- and 64-channel montages in source reconstruction. To further evaluate whether lower channel density distorts source estimates, we re‑ran the identical source‑

reconstruction pipeline on a 32-channel subset of the original 64-channel dataset [1] and extracted source power for the same 12 ROIs (e.g., mPFC, bilateral DLPFC, bilateral SM1). Correspondence was assessed at two levels: trial-wise source powers entered into Bayesian LMMs (powers extracted from 64- and 32-channel montages are treated as dependent and independent variables respectively) and between-participant correlations. Both analyses showed high concordance (Bayesian LMMs: *Est*s ≥ 0.762, *P*ps = 1.00; between-participants correlations: *r*s ≥ 0.912, *p*s < 0.001), indicating the 32-channel montage produces reliable source-level estimates (Fig E).

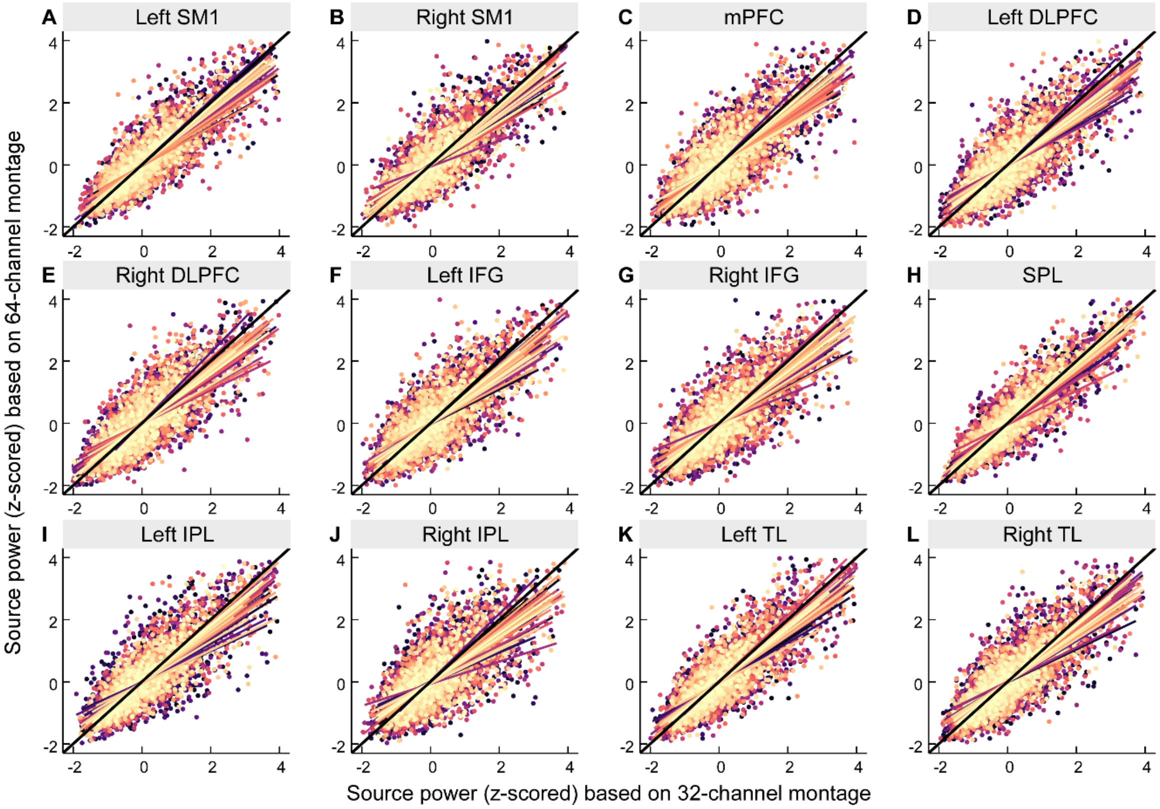

Fig E. Correspondence between source-power estimates derived from 32- and 64-channel montages in the Nickel et al 2022 [1]**.**

Panels show source power extracted from (A) Left primary sensorimotor cortex (SM1), (B) Right SM1, (C) medial prefrontal cortex (mPFC), (D) Left dorsolateral prefrontal cortex (DLPFC), (E) Right DLPFC, (F) Left inferior frontal gyrus (IFG), (G) Right IFG, (H) Superior parietal lobule (SPL), (I) Left inferior parietal lobule (IPL), (J) Right IPL, (K) Left temporal lobe (TL), (L) Right TL. Colored dots and lines depict individual-participant data and linear fits; the diagonal line indicates perfect correspondence.

Text G. Mega-analyses reinforced the expectation effects on pain processing

To enhance the robustness of our findings, we performed mega-analyses [6,7] integrating data from the current study and the independent dataset from Nickel et al 2022 [1]. Using Bayesian LMM, we examined how expectation parameters modulate pain perception across studies. The model accounted for stimulus intensity (LP = 1, HP = 2), expectation strength, precision, their interaction, and study origin as covariates, with random intercepts for participants:

DVs ~ Stimulus intensity + Expectation strength × Precision + Study + (1 | Participant)

G1. Pain ratings. The cross-study analysis yielded three main findings on the pain ratings (Fig F). First, we observed robust main effects across pooled datasets, with HP stimuli increasing subjective ratings (*Est* = 17.74, 95% HPD = [23.16, 26.45], *P*p = 1.00), expectation strength enhancing pain perception (*Est* = 24.81, 95% HPD = [19.66, 24.62], *P*p = 1.00), and heightened precision amplifying nociceptive responses (*Est* = 3.71, 95% HPD = [1.89, 5.51], *P*p = 1.00). Second, a critical interaction emerged between expectation strength and precision (*Est* = –7.42, 95% HPD = [–10.88, –3.91], *P*p = 1.00), demonstrating that expectation-driven pain modulation was more potent under low-precision conditions (–1 SD) compared to high-precision states (+1 SD). Notably, these effects remained stable after controlling for inter-study variability through explicit modeling of dataset origin, with Bayesian evidence thresholds (all *P*p = 1.00) confirming decisive support across all parameters.

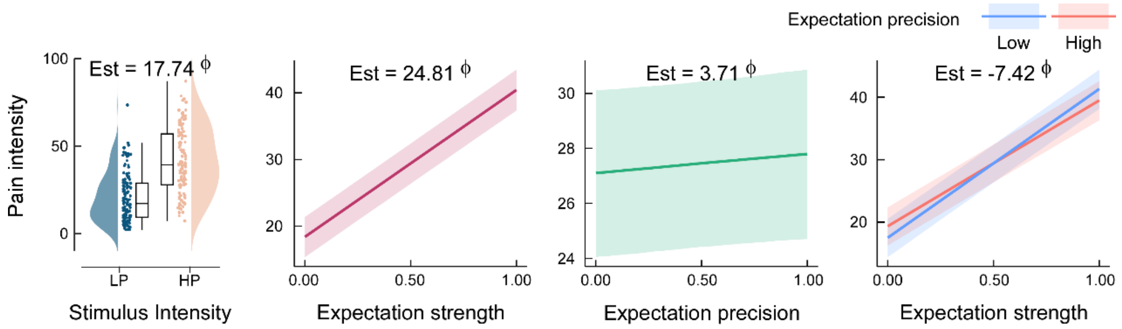

Fig F. Predictive effects on pain ratings.
Bayesian LMMs identified predictors of pain intensity ratings: stimulation intensity, expectation strength, and expectation precision, with a significant strength-precision interaction. Shaded bands: 95% prediction intervals. Violin plots: Individual data distributions (dots) with boxplots (median, IQR). Φ: Posterior probability > 97.5% and 95% HPD excludes 0. Data supporting this figure are available at https://doi.org/10.5281/zenodo.18503056.

G2. Laser evoked potentials. Following the established peak-trough method used in Nickel et al 2022 [1], N2 and P2 component amplitudes were quantified at Cz electrode by averaging trial-wise amplitudes within 30-ms windows centered at individual peak latencies for each participant.

Bayesian LMM employing the same analytical framework used for behavioral data, were applied to combined LEP datasets. As shown in Fig G, both stimulus intensity and expectation strength demonstrated robust fixed effects on LEP components. Specifically, HP stimuli elicited significantly larger N2 amplitudes (*Est* = –7.31, 95% HPD = [–7.68, –6.94], *P*p = 1.00) and P2 amplitudes (*Est* = 6.08, 95% HPD = [5.71, 6.46], *P*p = 1.00) compared to LP stimuli. Similarly, trials with stronger pain expectations showed enhanced N2 (*Est* = –8.04, 95% HPD = [–9.35, –6.72], *P*p = 1.00) and P2 amplitudes (*Est* = 6.62, 95% HPD = [5.35, 7.90], *P*p = 1.00). Notably, while expectation precision showed a marginal association with N2 amplitude reduction (*Est* = 1.25, 95% HPD = [-0.20, 2.70], *P*p = 0.95), no significant precision effect emerged for P2 components (*Est* = –0.36, 95% HPD = [–1.75, 1.06], *P*p = 0.70). These convergent findings across independent datasets confirm that both bottom-up nociceptive signaling (indexed by stimulus intensity) and top-down predictive processing (reflected in expectation strength) jointly modulate the N2-P2 complex, replicating and extending our time-domain ERP results reported in the main text.

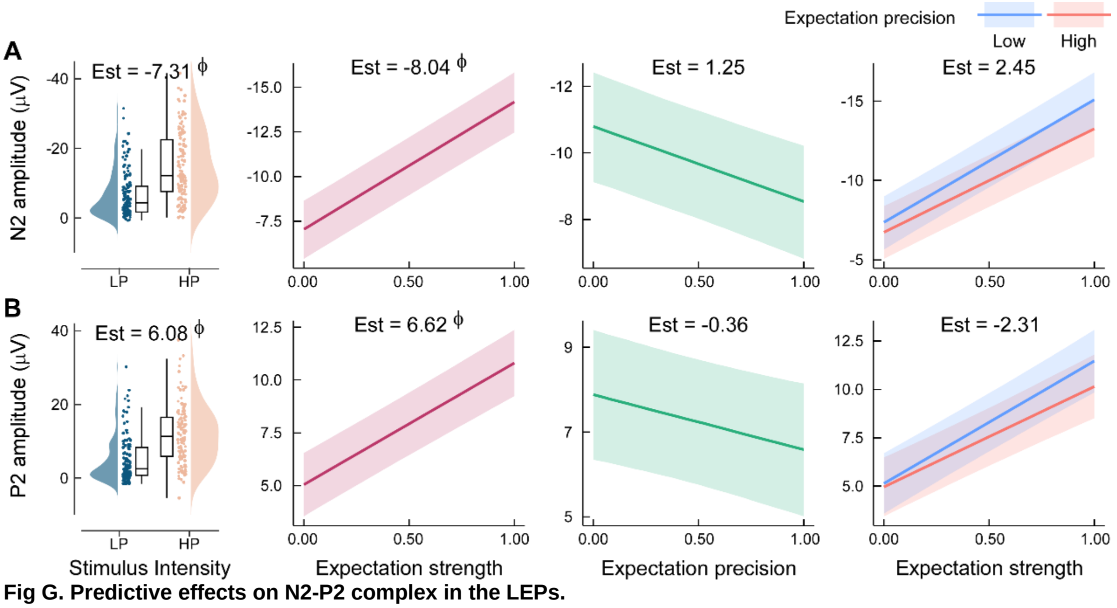

Fig G. Predictive effects on N2-P2 complex in the LEPs.
Bayesian LMMs revealed significant main effects of stimulation intensity and expectation strength on the amplitudes of both N2 and P2 components. Shaded bands: 95% prediction intervals. Violin plots: Individual data distributions (dots) with

boxplots (median, IQR). Φ: Posterior probability > 97.5% and HPD excludes 0. Data supporting this figure are available at https://doi.org/10.5281/zenodo.18503056.

G3. Laser induced EEG oscillations. To investigate pain-related neural dynamics, we characterized three distinct EEG responses within predefined spatiotemporal-frequency domains: 1) Laser-evoked potentials (LEP) at 1–10 Hz (100–400 ms) over frontocentral regions (FC1/FC2/Cz); 2) Alpha event-related desynchronization (α-ERD) at 7–13 Hz (500–900 ms) across parietal electrodes (P1/Pz/P2); 3) Gamma event-related synchronization (γ-ERS) at 60–90 Hz (200–350 ms) over frontocentral channels (FC1/FC2/Cz). Oscillation magnitudes were quantified by averaging spectral power within their respective temporal, spectral, and spatial boundaries.

As shown in Fig H, Bayesian linear mixed models revealed three key findings. First, significant stimulus intensity effects were observed for LEP (*Est* = 1.37, 95% HPD = [1.26, 1.49], *P*p = 1.00), α-ERD (*Est* = –0.20, 95% HPD = [–0.30, –0.10], *P*p = 1.00), and γ-ERS (*Est* = 0.011, 95% HPD = [0.005, 0.017], *P*p = 1.00), with high-pain trials showing enhanced responses. Second, expectation strength positively correlated with LEP (*Est* = 2.38, 95% HPD = [1.99, 2.79], *P*p = 1.00) and γ-ERS magnitudes (*Est* = 0.03, 95% HPD = [0.01, 0.05], *P*p = 1.00). Third, expectation precision exhibited an inverse relationship with LEP amplitude (*Est* = –0.69, 95% HPD = [–1.13, –0.26], *P*p = 1.00). Notably, no significant expectation-related modulation was detected for α-ERD (all *P*p < 0.95). These replicated findings using combined datasets confirm that both bottom-up nociceptive input (stimulus intensity) and top-down predictive signals (expectation strength/precision) differentially regulate pain-induced oscillatory activity, corroborating our principal conclusions.

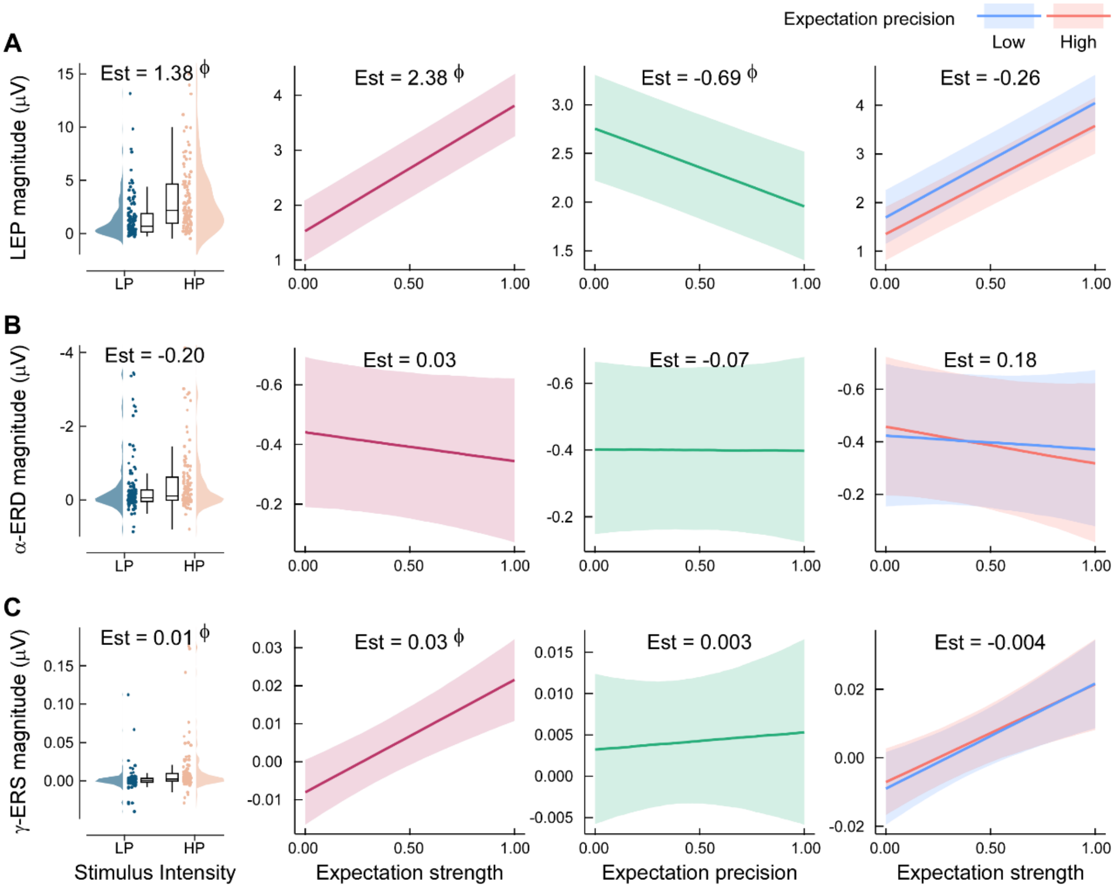

Fig H. Predictive effects on laser induced EEG oscillations.
Bayesian LMM models analyzing stimulus intensity, expectation strength, and precision effects LEP (A), α-ERD (B), and γ-ERS (C) magnitudes. Shaded bands: 95% prediction intervals. Violin plots: Individual data distributions (dots) with boxplots (median, IQR). Φ: Posterior probability > 97.5% and 95% HPD excludes 0. Data supporting this figure are available at https://doi.org/10.5281/zenodo.18503056.

G4. Anticipatory EEG oscillations. Building upon our core discovery of expectation strength modulating centro-parietal θ (4–7 Hz) and fronto-central α (7–13 Hz) oscillations, and precision regulating frontal β (13–30 Hz) and contralateral sensorimotor α oscillations, we validated these patterns in the merged dataset. Bayesian LMM revealed: (1) Expectation strength suppressed centro-parietal θ (*Est* = –0.08, 95% HPD = [–0.14, –0.02], *P*p = 0.99) and fronto-central α power (*Est* = –0.23, 95% HPD = [–0.32, –0.14], *P*p = 1.00). Expectation precision enhanced contralateral sensorimotor α (*Est* = 0.065, 95% HPD = [0.002, 0.13], *P*p = 0.98) and frontal β power (*Est* = 0.03, 95% HPD = [0.02, 0.04], *P*p = 1.00). These results (Fig I) precisely replicate our main findings, confirming the dissociable roles of expectation strength and precision in anticipatory dynamics.

Mediation analyses identified two critical pathways (Fig I). Both pathways demonstrate how anticipatory oscillations biologically instantiate predictive coding operations prior to nociceptive input.

- Fronto-central α mediation: Higher expectation strength was associated with decreased fronto-central α power, which in turn led to an increase in LEP oscillation magnitude (*indirect effect = 0.003, p* < 0.001, FDR corrected).
- Sensorimotor α mediation: Higher expectation precision was associated with enhanced contralateral sensorimotor α power, which in turn led to a decrease in LEP oscillation magnitude (indirect effect = –0.001, *p* = 0.03, FDR corrected).

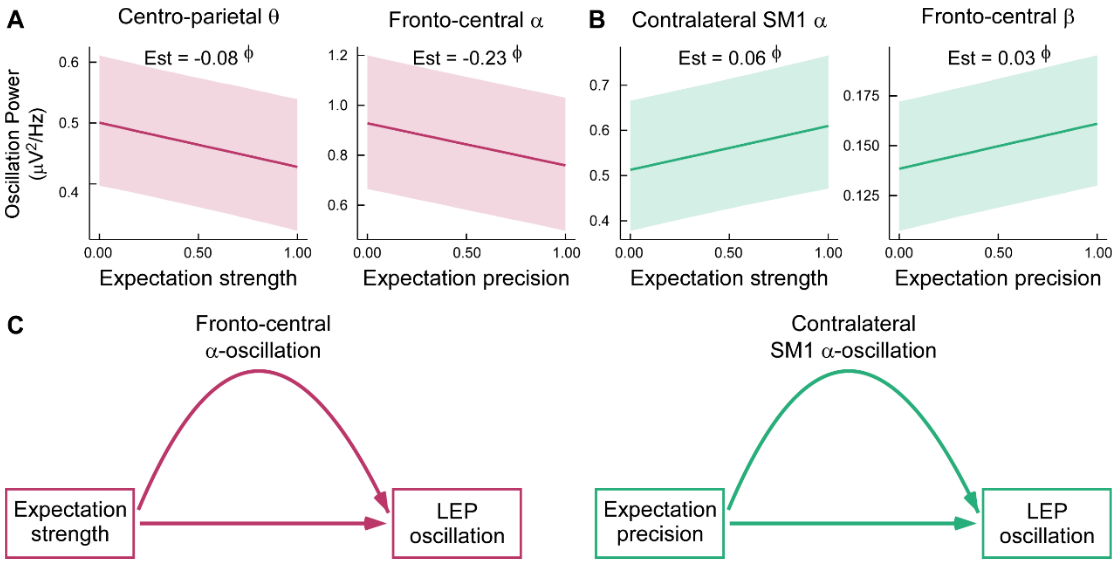

Fig I. Predictive effects on anticipatory EEG oscillations.
(A−B) Bayesian linear mixed models revealed distinct modulation patterns in preparatory oscillations: Expectation strength significantly suppressed centro-parietal θ and fronto-central α power, whereas expectation precision enhanced contralateral sensorimotor α and frontal β oscillations. Shaded bands: 95% prediction intervals. Φ: Posterior probability > 97.5% and HPD excludes 0. (C) Mediation models linking expectation, anticipatory EEG oscillation, and LEPs. Mediation analyses uncovered two oscillatory pathways shaping pain responses: Fronto-central α power mediated the transmission of expectation strength effects to laser-evoked potentials, while contralateral sensorimotor α power mediated precision-dependent gating of nociceptive signals. Data supporting this figure are available at https://doi.org/10.5281/zenodo.18503056.

SI References

1. Nickel MM, Tiemann L, Hohn VD, May ES, Gil Ávila C, Eippert F, et al. Temporal–spectral signaling of sensory information and expectations in the cerebral processing of pain. Proc Natl Acad Sci USA. 2022;119: e2116616119. doi:10.1073/pnas.2116616119

2. Rouder JN, Morey RD. Default Bayes Factors for Model Selection in Regression. Multivariate Behavioral Research. 2012;47: 877–903. doi:10.1080/00273171.2012.734737

3. Jepma M, Koban L, Van Doorn J, Jones M, Wager TD. Behavioural and neural evidence for self-reinforcing expectancy effects on pain. Nat Hum Behav. 2018;2: 838–855. doi:10.1038/s41562-018-0455-8

4. Daunizeau J, Adam V, Rigoux L. VBA: A Probabilistic Treatment of Nonlinear Models for Neurobiological and Behavioural Data. Prlic A, editor. PLoS Comput Biol. 2014;10: e1003441. doi:10.1371/journal.pcbi.1003441

5. Tzourio-Mazoyer N, Landeau B, Papathanassiou D, Crivello F, Etard O, Delcroix N, et al. Automated Anatomical Labeling of Activations in SPM Using a Macroscopic Anatomical Parcellation of the MNI MRI Single-Subject Brain. NeuroImage. 2002;15: 273–289. doi:10.1006/nimg.2001.0978

6. Eisenhauer JG. Meta ‐ analysis and mega ‐ analysis: A simple introduction. Teaching Statistics. 2021;43: 21–27. doi:10.1111/test.12242

7. Zugman A, Harrewijn A, Cardinale EM, Zwiebel H, Freitag GF, Werwath KE, et al. Mega ‐ analysis methods in ENIGMA : The experience of the generalized anxiety disorder working group. Human Brain Mapping. 2022;43: 255–277. doi:10.1002/hbm.25096
